# BLIMP-1-dependent differentiation of T follicular helper cells into Foxp3^+^ T regulatory type 1 cells

**DOI:** 10.3389/fimmu.2025.1519780

**Published:** 2025-02-24

**Authors:** Josep Garnica, Jun Yamanouchi, Robert Clarke, Joel Moro, Shari Thiessen, Javier Montaño, Debajyoti Mondal, Pau Serra, Pere Santamaria

**Affiliations:** ^1^ Institut D’Investigacions Biomèdiques August Pi i Sunyer, Barcelona, Spain; ^2^ Department of Microbiology, Immunology and Infectious Diseases, Snyder Institute for Chronic Diseases, Cumming School of Medicine, University of Calgary, Calgary, AB, Canada

**Keywords:** peptide-major histocompatibility complex (pMHC), nanomedicine, T-follicular helper cells, T-regulatory type-1 cells, Foxp3^+^ T-regulatory type-1 cells, BLIMP-1, type 1 diabetes (T1D), experimental autoimmune encephalomyelitis (EAE)

## Abstract

T-regulatory-type-1 (TR1) cells are a subset of interleukin-10-producing but Foxp3^–^ Treg cells that arise in response to chronic antigenic stimulation. We have shown that systemic delivery of autoimmune disease-relevant peptide-major histocompatibility complex class II (pMHCII)-coated nanoparticles (pMHCII-NP) triggers the formation of large pools of disease-suppressing Foxp3^–^ TR1 cells from cognate T-follicular helper (TFH) cell precursors. Here we show that, upon treatment withdrawal, these Foxp3^–^ TR1 cells spontaneously differentiate into a novel immunoregulatory Foxp3^+^ TR1 subset that inherits epigenetic and transcriptional hallmarks of their precursors, including clonotypic T-cell receptors, and is distinct from other Foxp3^+^ Treg subsets. Whereas the transcription factor BLIMP-1 is dispensable for development of conventional Foxp3^+^ Treg cells, it is necessary for development of Foxp3^+^ TR1 cells. In a model of central nervous system autoimmunity, abrogation of BLIMP-1 or IL-10 expression in the Foxp3^–^ and/or Foxp3^+^ TR1 subsets inhibits their development or anti-encephalitogenic activity. Thus, the TFH-TR1 transdifferentiation pathway results in the generation of two distinct autoimmune disease-suppressing, IL-10-producing TR1 subsets that are distinguished by the expression of Foxp3 and Foxp3 target genes.

## Introduction

The peripheral immune system harbors different types of regulatory T cells (Tregs). The most prevalent and best understood CD4^+^ Treg cell subset expresses the transcription factor Foxp3 (Forkhead box P3) and the high-affinity receptor for Interleukin 2 (IL-2; CD25). Tregs can be broadly classified into thymic (natural) and peripheral (adaptive) Treg cells (1). Whereas thymic Treg cells largely arise in response to high avidity interactions between developing thymocytes and thymic APCs, peripheral Treg cells arise from conventional naïve CD4^+^ T cells upon antigenic stimulation in the presence of cytokines such as TGFβ (transforming growth factor - beta) and IL-2 (2). Both Treg cell types express high levels of CD25, CTLA-4 (Cytotoxic T-Lymphocyte Antigen 4), CD39, CD73, LAG-3 (Lymphocyte-activation gene 3), TIGIT (T Cell Immunoreceptor with Ig And ITIM Domains) and secrete various immunoregulatory cytokines such as IL-10, TGFβ and IL-35 (3).

Apart from the classical Foxp3^+^ Treg cell subsets, several other IL-10-producing and phenotypically heterogeneous, but Foxp3^–^, subsets of Treg cells have been described. T-regulatory type 1 (TR1) cells are one of these subsets (4). Both, Foxp3^–^ TR1 and Foxp3^+^ Treg cells have similar cytokine secretion profiles and share phenotypic properties. For example, they both secrete IL-10, TGFβ and IL-35, and co-express the immunoregulatory molecules CTLA-4, PD-1 (Programmed death-1), ICOS (Inducible T-cell costimulator), CD39, CD73 and granzyme B, among others (5, 6). Despite these phenotypic and functional similarities, the developmental biology of these two Treg cell subsets is regulated by different transcription factors. Whereas Foxp3 is required for Foxp3^+^ Treg but not TR1 cell development, BLIMP-1 (B lymphocyte-induced maturation protein 1), encoded by the Positive Regulatory Domain 1 (*Prdm1*) gene, is required for TR1 but not Foxp3^+^ Treg cell development (7, 8). Although Foxp3^+^ Treg cells (like TR1 cells) express both BLIMP-1 and IRF4, which coordinately promote *Il10* expression, specific deletion of *Prdm1* in T cells not only does not abrogate, but rather promotes Foxp3^+^ Treg cell formation (7).

Treatment of wild-type mice with nanoparticles (NPs) coated with mono-specific autoimmune disease-relevant peptide-major histocompatibility complex class II (pMHCII) molecules (9) can resolve inflammation in various organ-specific autoimmune disease models in a disease-specific manner without impairing normal immunity (10–12). pMHCII-NP therapy functions by systemically expanding and then re-programming cognate T follicular helper cells (TFH) into expanded pools of oligoclonal TR1 cells (7, 13). This transdifferentiation process evolves through a transitional *Il10^–^Cxcr5^low^Ccr5^–^Prdm1^–^Bcl6^low^* TR1-like cell stage. *De novo* expression of *Pdrm1* in this transitional TR1-like cell subset, upon the loss of *Bcl6* and *Tcf7* expression during the TFH-to-TR1-like cell conversion, results in terminal differentiation of these cells into immunoregulatory *Il10^+^Cxcr5^–^Ccr5^+^Prdm1^+^Bcl6^–^* TR1 cells (7). Importantly, whereas deletion of *Prdm1* abrogates the TR1-like to TR1 cell conversion, it increases the peripheral frequency of Foxp3^+^/CD25^+^ T cells, demonstrating that this transcription factor is necessary for development of the former, but dispensable for development of the latter (7). Additional work has shown that the TFH-to-TR1 cell transition is accompanied by both, downregulation of TFH cell-specific gene expression due to loss of chromatin accessibility, and upregulation of TR1 cell-specific genes linked to chromatin regions that remain accessible throughout the transdifferentiation process, with minimal generation of new open chromatin regions. Notably, most of the genes linked to these regions of the chromatin that remain open in TR1 cells, including *Il10*, are already poised for expression at the TFH cell stage: whereas these genes are closed and hypermethylated in Tconv cells, they are accessible, hypomethylated and enriched for H3K27ac-marked and hypomethylated active enhancers in TFH cells (14).

Here, we show that pMHCII-NP-derived TR1 cells arising from TFH cells spontaneously differentiate *in vivo* into a novel immunoregulatory Foxp3^+^ TR1-like subset that inherits the transcriptional and epigenetic hallmarks of its Foxp3^–^ TR1 precursors and is transcriptionally and developmentally distinct from other Foxp3^+^ Treg cell subsets described to date, including T-follicular regulatory (TFR) cells.

## Results

### A pMHCII-NP-induced TR1 subset that expresses Foxp3

We set out to investigate the fate of pMHCII-NP-induced splenic tetramer^+^ (Tet) CD4^+^ T cells upon treatment withdrawal. This was done by comparing absolute and relative numbers and transcriptional profiles of the various BDC2.5mi/IA^g7^ Tet^+^ subsets arising in pre-diabetic NOD mice in response to BDC2.5mi/IA^g7^-NP therapy (twice a week for 5 weeks), both shortly after treatment completion (3 days after the last dose; referred to as “week 0”), or 5 and 10 weeks later (“week 5” and “week 10”). BDC2.5mi/I-A^g7^-specific CD4^+^ T cells comprise a population of autoreactive T cells that contribute to the progression of spontaneous autoimmune diabetes in NOD mice. The size of this type 1 diabetes-relevant T cell specificity is small and barely detectable in untreated NOD mice, but treatment with cognate pMHCII-NPs leads to the expansion and formation of anti-diabetogenic TR1 cells that retain the antigenic specificity of their precursors. As a result, treatment of hyperglycemic NOD mice with these compounds results in the reversal of type 1 diabetes (10).

**Figure 1:**
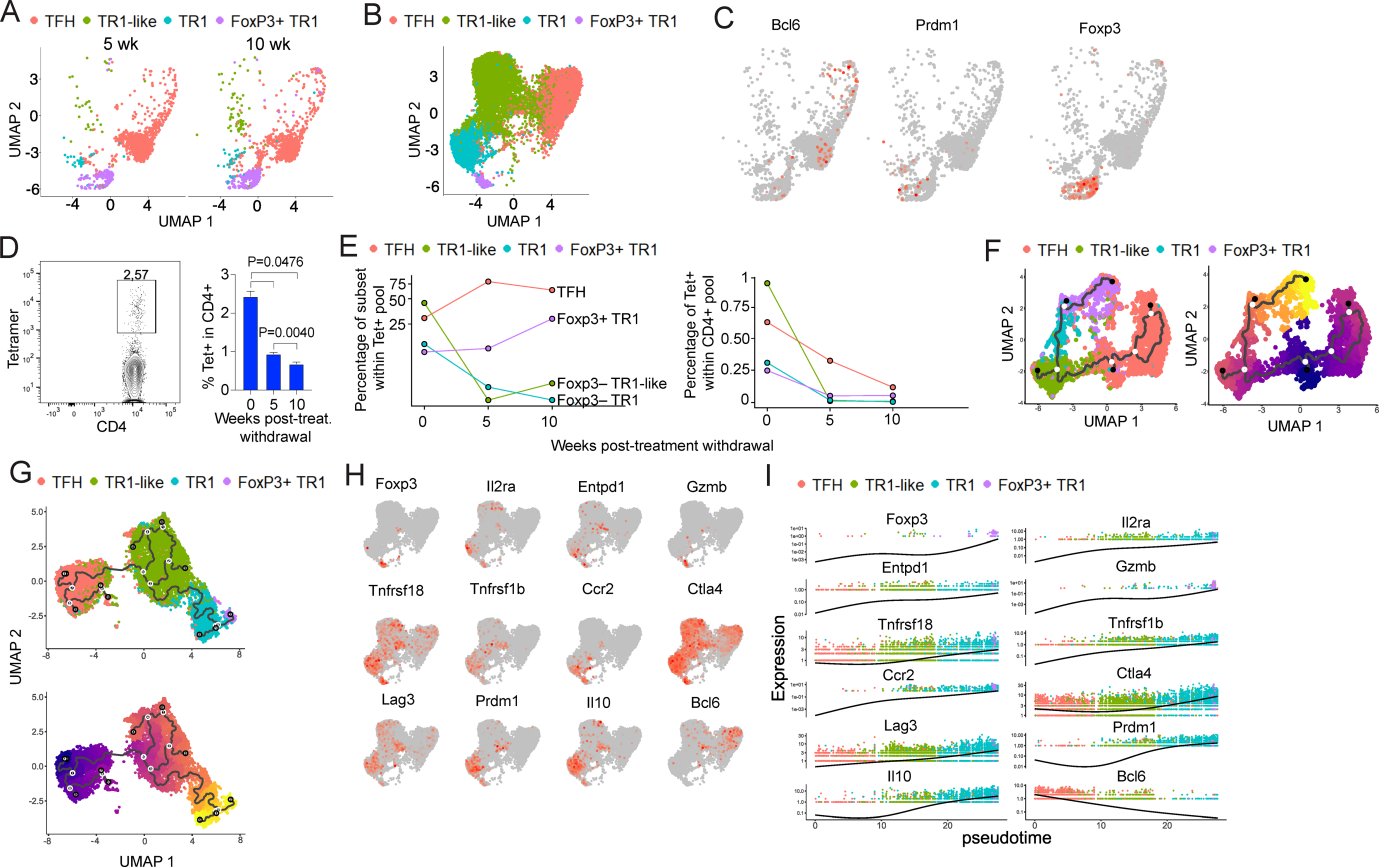
Dynamic evolution and transcriptional changes of the pMHCII-NP-induced tetramer^+^ T cell subsets post-treatment withdrawal leading to formation of a cognate Foxp3^+^ TR1 cell subset. **(A)** UMAP visualization of scRNAseq data corresponding to BDC2.5mi/I-A^g7^-NP-induced splenic tetramer^+^ cells from female NOD mice 5 and 10 weeks after the last dose of pMHCII-NPs. False tetramer-positive cells (*Maf^–^Ccr7^+^Sell^+^*) were excluded from analysis. **(B)** UMAP representation of scRNAseq data for BDC2.5mi/I-A^g7^-NP-induced Tet^+^ cells isolated from spleen of treated female mice (n=3) at the end of treatment (week 0). Cells cluster into Tet^+^ TFH (red), TR1-like (green) and TR1 (blue) subsets (all Foxp3^–^) and a small Foxp3^+^ TR1-like subset (purple). **(C)** Feature plot of UMAP representation of BDC2.5mi/I-A^g7^-NP-induced tetramer^+^ cells corresponding to pooled data from weeks 5- and 10-weeks withdrawal cells from **(A)**. Representative transcription factor genes for TFH (*Bcl6*), Foxp3^–^ TR1 (*Prdm1*), and Foxp3^+^ TR1 cells (*Foxp3*) are shown. **(D)** Representative tetramer^+^ and CD4 staining profiles of splenic CD4^+^ T cells at the end of treatment (left) and average percentages of tetramer^+^ CD4^+^ T cells at different times after treatment withdrawal. Data corresponds to n=3 female NOD mice for each time point. **(E)** Line plots representing the relative proportion of each subset within the total splenic tetramer^+^ T cell pool (left), or the changes in the absolute percentages of the various tetramer^+^ subsets within the total splenic CD4^+^ T cell pool as a function of time post-treatment withdrawal (right). **(F)** Monocle3-based UMAP visualization of pooled transcriptomic data for week 0, week 5 and week 10 tetramer^+^ cells. Left panel annotates the Tet^+^ TFH, TR1-like, Foxp3^–^ TR1 and Foxp3^+^ TR1 subsets. The right panel shows the predicted cell differentiation trajectory when setting week 0 Tet^+^ TFH cell pool as the origin of pseudotime. **(G)** Monocle3-based UMAP visualization of the various Tet^+^ sub-clusters from **(F)** (top) and cell trajectory prediction when setting the Tet^+^ TFH sub-pool as the origin of pseudotime (bottom). **(H)** Feature UMAP plots for representative Treg-associated genes in the BDC2.5mi/I-A^g7^-NP-induced tetramer^+^ cells from the mice in **(B)**. **(I)** Scaled single-cell expression of Treg-associated markers from **(G)** over pseudotime. Dots representing cells are colored based on clustering in **(B)**. Only cells with more than one count are shown. The black lines from left to right correspond to the mean of gene expression over the pseudotime.

As expected, scRNAseq analysis of the pMHCII-NP-induced Tet^+^ T cell pool at week 0 largely replicated previous results (7), yielding three well-defined sub-clusters: TFH, TR1-like and TR1. Surprisingly, however, the splenic Tet^+^ T cell pools that were obtained at weeks 5 and 10 post-treatment withdrawal revealed the appearance and progressive accumulation of an additional Tet^+^ T cell subset that expressed *Foxp3*, in association with a significant reduction in the relative size of the TR1-like and TR1 sub-pools (**Figure 1A**). Re-examination of previous week 0 pMHCII-NP-induced Tet^+^ T cell datasets confirmed the consistent presence of a very small subset of Tet^+^ Foxp3^+^ cells that lied close to the terminally differentiated TR1 cell sub-pool in UMAP embedding (**Figure 1B**). Although the various sub-clusters of Tet^+^ cells present in the spleen 10 weeks post-treatment withdrawal had undergone transcriptional changes with time (see further below), they retained the expression of expected sub-pool-specific genes, such as *Bcl6* (TFH sub-cluster), *Prdm1* (Foxp3^–^ TR1 sub-cluster) and Foxp3 (Foxp3^+^ sub-cluster) (**Figure 1C**), consistent with the corresponding cell subset annotations (7).

There was a significant reduction in the overall size of the total pMHCII-NP-induced splenic Tet^+^ T cell pool at weeks 5 and 10 post-treatment withdrawal relative to its week 0 counterpart (**Figure 1D**). This reduction impacted the four different Tet^+^ subsets differently (**Figure 1E**). Specifically, although the absolute number of Tet^+^ TFH cells that persisted in the spleen by weeks 5 and 10 post-treatment withdrawal had progressively declined to about one third of the original number (**Figure 1E**, right), the relative size of the Tet^+^ TFH cell pool increased by ~3-4 fold during this period (**Figure 1E**, left). Likewise, there was a ~50% reduction in the absolute numbers of Tet^+^ Foxp3^+^ cells over this time-period, but a 3-fold increase in the relative size of this Tet^+^ subset (**Figure 1E**). These changes were accompanied by a significant decay (~10-fold) in both the relative and absolute numbers of the splenic Tet^+^ TR1-like and Tet^+^ Foxp3^–^ TR1 subsets (**Figure 1E**).

When taken together, these data suggested that, upon pMHCII-NP treatment withdrawal, a fraction of the Tet^+^ Foxp3^–^ TR1-like and TR1 cells that arise in response to pMHCII-NP encounters exit the spleen (or perish), whereas the spleen-resident fraction continues to differentiate into a Tet^+^ Foxp3^+^ subset, leading to a progressive increase in its relative size. This interpretation of the data was supported by pseudotime trajectory analysis. As shown in **Figure 1F**, all the Tet^+^ cell clusters that were found at the different time points (weeks 0, 5 and 10) were connected by a gene expression gradient. Specifically, when we set the starting point of pseudotime at the week 0’s TFH cluster (experimentally established to be the cell precursors of the TFH-TR1 pathway) (7), pseudotime trajectory analysis generated a pathway that involves progressive differentiation of these cells into Foxp3^–^ TR1-like and Foxp3^–^ TR1 cells, and ultimately, Foxp3^+^ cells (**Figure 1F**). We hereinafter refer to these cells as Foxp3^+^ TR1 cells.

### The pMHCII-NP-induced T cell subsets undergo transcriptional changes upon treatment withdrawal but retain their lineage identity

To more precisely define the transcriptional make-up of the Tet^+^ Foxp3^+^ TR1 subset, and to ascertain whether the spleen-resident Tet^+^ TFH and Tet^+^ Foxp3^+^ TR1 subsets are transcriptionally stable, we compared the transcriptional profiles of the week 5 and 10 subsets to their week 0 counterparts, via scRNAseq. Since the week 5 and week 10 subsets were essentially identical (only 5 differentially expressed genes/cell type (|log2FC| > 0.5) (**Supplementary Figure 1A**; **Supplementary Data Sheet 1**), we pooled them for comparisons to the week 0 subsets.

The week 5/10 Tet^+^ TFH cells displayed 128 differentially expressed genes as compared to the week 0 Tet^+^ TFH cells (|log2FC| > 0.5). Most of these genes (n=92/128; 71.8%), including many TFH-relevant genes, such as *Ascl2, Bcl6, Cebpa, Cxcr5, Id3, Il21, Maf, Pdcd1, Pou2af1, and Tox2*, were downregulated (**Supplementary Figure 1B**, top, and **Supplementary Data Sheet 2**). The remaining 36 genes (28.1%) were significantly upregulated, most notably *Il7r* (**Supplementary Figure 1B**, top, and **Supplementary Data Sheet 2**). Likewise, the week 5/10 Foxp3^+^ TR1 sub-pool showed a total of 153 differentially expressed genes (|log2FC| > 0.5), most of which were also downregulated (n=109/153; 71.2%). This list included the TR1 genes *Ahr*, *Ccr5, Entpd1, Icos, Id2, Ifng, Il10, Havcr2, Lag3, Maf, Prdm1 and Tnfrsf18*, and some of the genes that TR1 cells upregulate when they presumably transition from Foxp3^–^ to Foxp3^+^ TR1 cells (see below), such as *Ccl4, Ccl5, Ccr2, Cxcr6 and Gzmb* (**Supplementary Figure 1B**, bottom, and **Supplementary Data Sheet 2**). The remaining 44/153 genes (28.7%) were upregulated, most notably the TFH transcription factor-coding gene *Tcf7*, the anti-apoptotic *Bcl2* gene, and the gene encoding the lymphoid tissue egress receptor *S1pr1* (**Supplementary Figure 1B**, bottom, and **Supplementary Data Sheet 2**). Together, these results suggest that the Tet^+^ TFH and Foxp3^+^ TR1 subsets, unlike their Foxp3^–^ TR1-like and Foxp3^–^ TR1 counterparts, persist in the spleen long-term post-treatment withdrawal, attaining a memory TFH-like transcriptional profile or a “resting” transcriptional profile with downregulation of genes associated with the cells’ immunosuppressive, trafficking and cell adhesion properties, respectively (15–17).

Collectively, these observations suggested that the TFH-to-TR1 transdifferentiation pathway involves the differentiation of Foxp3^–^ TR1-like and Foxp3^–^ TR1 cells into a TR1 subset that expresses Foxp3.

### Identity of the pMHCII-NP-induced Foxp3^+^ TR1 cell subset

Detailed transcriptomic analyses of the small cluster of Foxp3^+^ T cells contained within the pMHCII-NP-induced Tet^+^ pool at week 0 suggested that this T cell subset is developmentally related to the previously described TR1 subset (**Figure 1B**). As also seen in the trajectory analyses of the pooled Tet^+^ cell subsets found at weeks 0, 5 and 10 post-treatment withdrawal, trajectory analysis of the week 0 Tet^+^ cells suggested that such Foxp3^+^ TR1 cells are another cellular component of the TFH-to-TR1 transdifferentiation pathway. Pseudotime inference, when setting TFH cells as the origin, situated the Tet^+^ Foxp3^+^ TR1 sub-pool at the end of the pathway, emerging from its Foxp3^–^ TR1 counterpart (**Figure 1G**). In addition to expressing high levels of the Foxp3^+^ Treg cell-associated genes *Foxp3*, *Il2ra* and *Gzmb*, these cells also expressed high levels of other TR1 cell-associated markers such as *Ccr2, Ctla4, Entpd1, Il10*, *Lag3*, *Tnfrsf18* and *Tnfrsf1b*, but not TFH markers such as *Bcl6* (**Figure 1H**). In fact, all these genes progressively increased their expression levels along the pathway, as the pMHCII-NP-cognate Tet^+^ TFH cells progressively acquired TR1-like and TR1 gene expression profiles, peaking at the Foxp3^+^ TR1 cell stage (**Figure 1I**).

**Figure 2:**
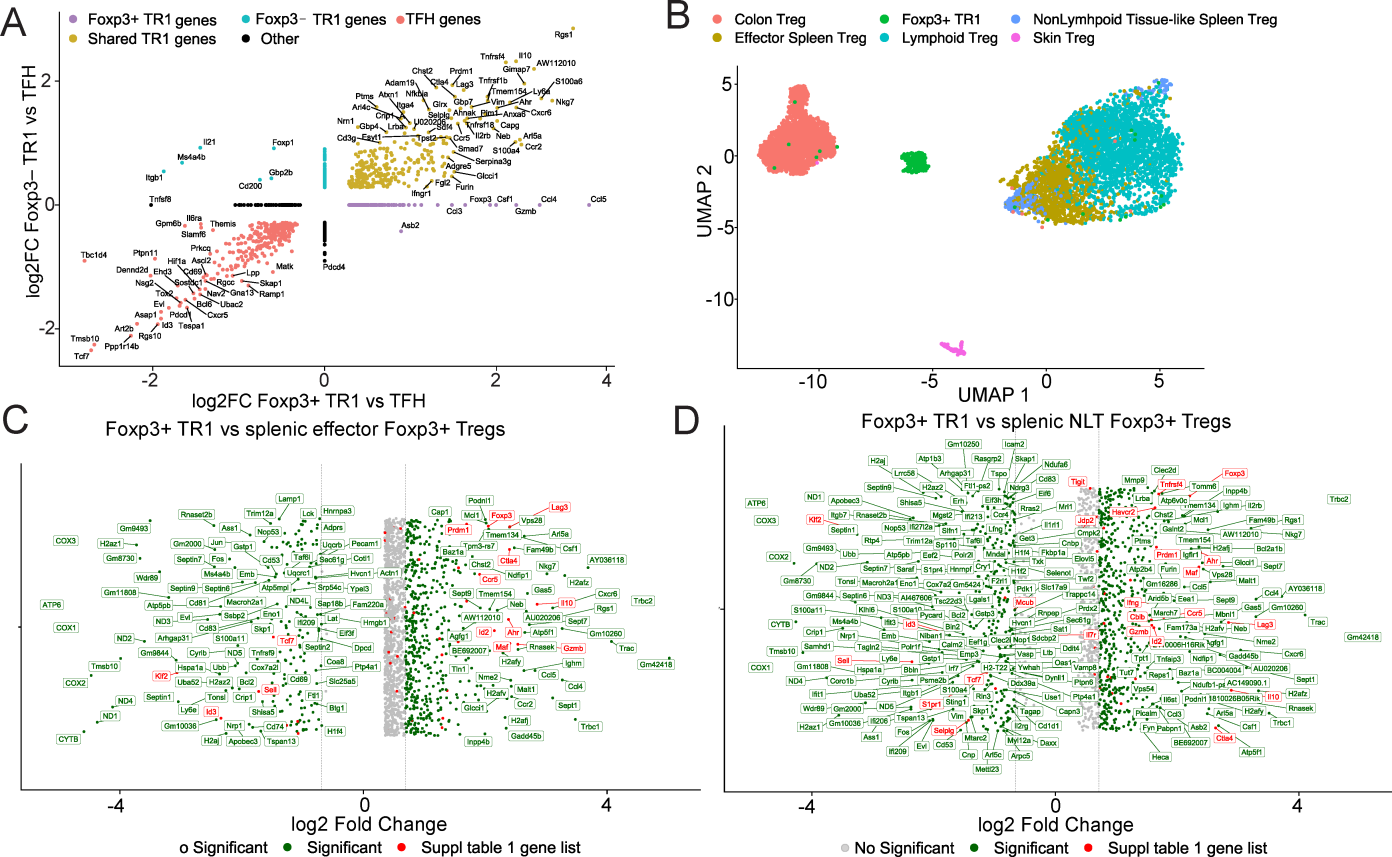
Comparison of the Tet^+^ Foxp3^+^ TR1 subset with Foxp3^–^ TR1 cells and other Foxp3^+^ Treg cell types. **(A)** Two-dimensional plot depicting the log2FC gene expression values for Tet^+^ Foxp3^+^ TR1 vs Tet^+^ TFH cells (x-axis) and Tet^+^ Foxp3^–^ TR1 vs Tet^+^ TFH cells (y-axis), excluding genes with an adjusted P > 0.05 (Wilcoxon test). Genes are colored based on whether they are differentially upregulated (lemon) or downregulated (red) in both subsets vs TFH cells (shared TR1 genes), only differentially upregulated in the Foxp3^+^ TR1 pool (purple; Foxp3^+^ TR1 genes), only differentially upregulated in the Foxp3^–^ TR1 pool (light blue; TR1 genes), or not fitting in any of the mentioned conditions (Other; black). All genes are annotated except when overlapping with 20 or more genes on the plot (**Supplementary Data Sheet 4**). **(B)** Batch-corrected UMAP visualization plot of BDC2.5mi/I-A^g7^-NP-induced Tet^+^ Foxp3^+^ TR1 cells and Foxp3^+^ Treg cell subsets from colon, skin, spleen (divided into effector Tregs and Non-lymphoid-like (NLT) Tregs) and lymph nodes (Lymphoid Tregs, pooled from mesenteric and brachial lymph nodes) (18). **(C, D)** Fold change plot of differential gene expression analysis using Wilcoxon test between Tet^+^ Foxp3^+^ TR1 and effector splenic Foxp3^+^ Tregs **(C)** or NLT splenic Foxp3^+^ Tregs **(D)**, respectively. Only genes with adjusted P < 0.05 and |log2FC| > 0.25 are shown. Genes with |log2FC| > 0.5 are shown in green, while genes with |log2FC| < 0.5 are displayed in grey. Genes listed in **Supplementary Table 1** are colored and annotated in red. Vertical lines correspond to the -0.5 and 0.5 values, respectively, of the log2FC scale. All genes are annotated except when overlapping with 20 or more genes on the plot (**Supplementary Data Sheet 3**).

Despite the high transcriptional similarities between the Foxp3^+^ TR1 and Foxp3^–^ TR1 subsets, differential gene expression analysis revealed a significant number of differences. Specifically, the Foxp3^+^ TR1 subset upregulated and downregulated 77 and 60 genes, respectively, as compared to its Foxp3^–^ counterpart (|log2FC| > 0.5). The fold-change plot shown on **Supplementary Figure 1C** (**Supplementary Data Sheet 3**) identifies the genes involved. The most significantly upregulated genes, in addition to *Foxp3*, include *Csf1, Gzmb, Il2ra, Mmp9, Nkg7*, and *Sdc4*, as well as genes encoding several chemokine ligands and receptors such as *Ccl3, Ccl4, Ccl5* and *Ccr2*. The most significantly downregulated genes in these Tet^+^ Foxp3^+^ TR1 cells, as compared to their Foxp3^–^ counterparts, included *Gpm6b, Itgb1, Ms4a4b* and *Tbc1d4.* Notably, the Foxp3^+^ TR1 subset further downregulated genes that, along the TFH-TR1 axis, are predominantly expressed at the TFH cell stage and then progressively and markedly downregulated at the Foxp3^–^ TR1-like and Foxp3^–^ TR1 cell stages. This list included *Il6ra, Il21, Foxp1, Tcf7* and *Tnfsf8*, to just name a few (**Supplementary Figure 1C**, **Supplementary Data Sheet 3**). Two-dimensional alignment of gene expression differences between Tet^+^ Foxp3^–^ TR1 vs. Tet^+^ TFH cells and Foxp3^+^ TR1 vs. Tet^+^ TFH cells (**Figure 2A**, **Supplementary Data Sheet 4**) confirmed that most of the TFH genes that were downregulated by the Foxp3^–^ TR1 subset as it evolved from the transitional TR1-like sub-pool, remained downregulated at the Foxp3^+^ TR1 stage. Likewise, many of the genes that were upregulated by Foxp3^–^ cells as compared to Tet^+^ TFH (and the transitional Tet^+^ TR1-like) cells, including *Ccr5, Ctla4, Lag3, Il10, and Prdm1*, remained upregulated at the Foxp3^+^ TR1 stage (**Figure 2A**, **Supplementary Data Sheet 4**).

To further explore the potential biological implications of the above gene expression differences, we conducted an overrepresentation analysis (ORA) using the Gene Ontology dataset (GO) and the genes that are significantly upregulated by Tet^+^ Foxp3^+^ TR1 cells as compared to their Foxp3^–^ TR1 counterparts. As shown in **Supplementary Figure 2** and **Supplementary Data Sheet 5**, the Foxp3^+^ TR1 subset overexpresses pathways associated with cell adhesion and migration, proliferation, and negative immune regulation. Collectively, these results provided additional support for the hypothesis that the Tet^+^ Foxp3^+^ TR1 subset arises from its Foxp3^–^ counterpart.

### pMHCII-NP-induced Foxp3^+^ TR1 cells are transcriptionally distinct from other Foxp3^+^ Treg cell subsets

We next compared the transcriptional profile of the pMHCII-NP-induced Foxp3^+^ TR1 subset to that of other Foxp3^+^ Treg cell subsets. We focused on published scRNAseq datasets corresponding to murine Foxp3^+^ Treg cells isolated from skin, colon, lymph nodes and spleen in steady-state conditions (18). **Figure 2B** shows a batch-corrected UMAP plot comparing pMHCII-NP-induced Tet^+^ Foxp3^+^ TR1 cells (harvested from the spleen) and the Foxp3^+^ Treg cell subsets described by Miragaia et al. (E-MTAB-6072 accession number in ref (18)) including the splenic effector Treg and the Non-Lymphoid Tissue (NLT)-like Treg cell subsets. Despite the fact that the Tet^+^ Foxp3^+^ TR1 subset arises in the spleen, it is clearly distinct from other splenic Treg cell subsets.

Head-to-head comparison of differential gene expression between the Tet^+^ Foxp3^+^ TR1 subset and splenic effector and NLT-like Foxp3^+^ Tregs indicated that the former exhibits a clearly distinct transcriptional signature (**Figures 2C, D**, **Supplementary Data Sheet 3**). Specifically, the Tet^+^ Foxp3^+^ TR1 cells upregulates 540 genes and downregulates 189 genes as compared to the splenic effector Treg cell subset and upregulates 581 and downregulates 330 genes as compared to the splenic NLT-like Treg cell subset (|log2FC| >0.5 and adjusted P value <0.05). In both cases, the Tet^+^ Foxp3^+^ TR1 subset expresses higher levels of *Ahr, Cblb, Ccr5, Ctla4, Foxp3, Gzmb, Havcr2, Id2, Il10, Ifng, Lag3, Maf* and *Prdm1*, among others, consistent with its TR1-like transcriptional make-up. It is noteworthy that the pMHCII-NP-induced Foxp3^+^ TR1 subset also expresses significantly higher levels of genes involved in cell migration and trafficking as compared to conventional splenic Treg cells, including *Ccl3, Ccl4, Ccl5* and *Cxcr6*. In addition, these Foxp3^+^ TR1 cells express lower levels of the spleen/lymph node homing ligands/receptor-coding genes *Ccr7, Sell* and *Selplg* (**Figures 2B–D**, **Supplementary Data Sheet 3**), suggesting that the Tet^+^ Foxp3^+^ TR1 subset is programmed to migrate to extra-lymphoid sites, presumably to effect immunoregulation. In contrast, the conventional Foxp3^+^ Treg cell types express much higher levels of the cyclooxygenase genes *Cox1, Cox2* and *Cox3*, involved in prostaglandin (PGE2) synthesis.

The similarities and differences among Foxp3^+^ TR1, Foxp3^–^ TR1 and other Foxp3^+^ Treg subsets become clearer when comparing heatmaps corresponding to transcription factor (**Supplementary Figure 3**) and cytokine/chemokine and cytokine/chemokine receptor profiles (**Supplementary Figure 4**). It is noteworthy that the Foxp3^+^ TR1 cells express much higher levels of *Foxp3* than their conventional Foxp3^+^ Treg counterparts. It is also worth noting that both the Foxp3^–^ TR1 and, in particular, the Foxp3^+^ TR1 subsets express high levels of the chemokine genes *Ccl3, Ccl4* and *Cxcl10*, which encode ligands for CCR5 and CXCR3, respectively, whose genes are also highly upregulated in these T cells as compared to conventional Foxp3^+^ Treg cells. These chemokine ligand and receptor pairs may help enhance the autocrine recruitment of Foxp3^–^ and Foxp3^+^ TR1 cells to sites of inflammation.

It is also important to note that these comparisons illustrate the existence of important transcriptional differences between the Foxp3^+^ TR1 subset and T-Follicular Regulatory (TFR) cells, which arise from Foxp3^+^ T cells. For example, the former does not express TFR-associated genes, such as *Bcl6* or *Cxcr5* and expresses genes that are silent in TFR cells such as *Ccr5*.

Taken together, the above results indicate that the pMHCII-NP-induced Foxp3^+^ TR1 subset is distinct from other Foxp3^+^ Treg cell subsets. Although also different than their hypothetical precursors (Tet^+^ Foxp3^–^ TR1 cells), these Foxp3^+^ TR1 cells retain a TR1-like transcriptomic signature, upregulate a significant number of genes involved in immune cell trafficking and adhesion, and express higher levels of genes associated with the immunosuppressive properties of both pMHCII-NP-induced Foxp3^–^ TR1 and conventional splenic Tregs.

### Recruitment of islet-specific Foxp3^–^ and Foxp3^+^ TR1 cells to pancreatic islets of NOD mice

**Figure 3:**
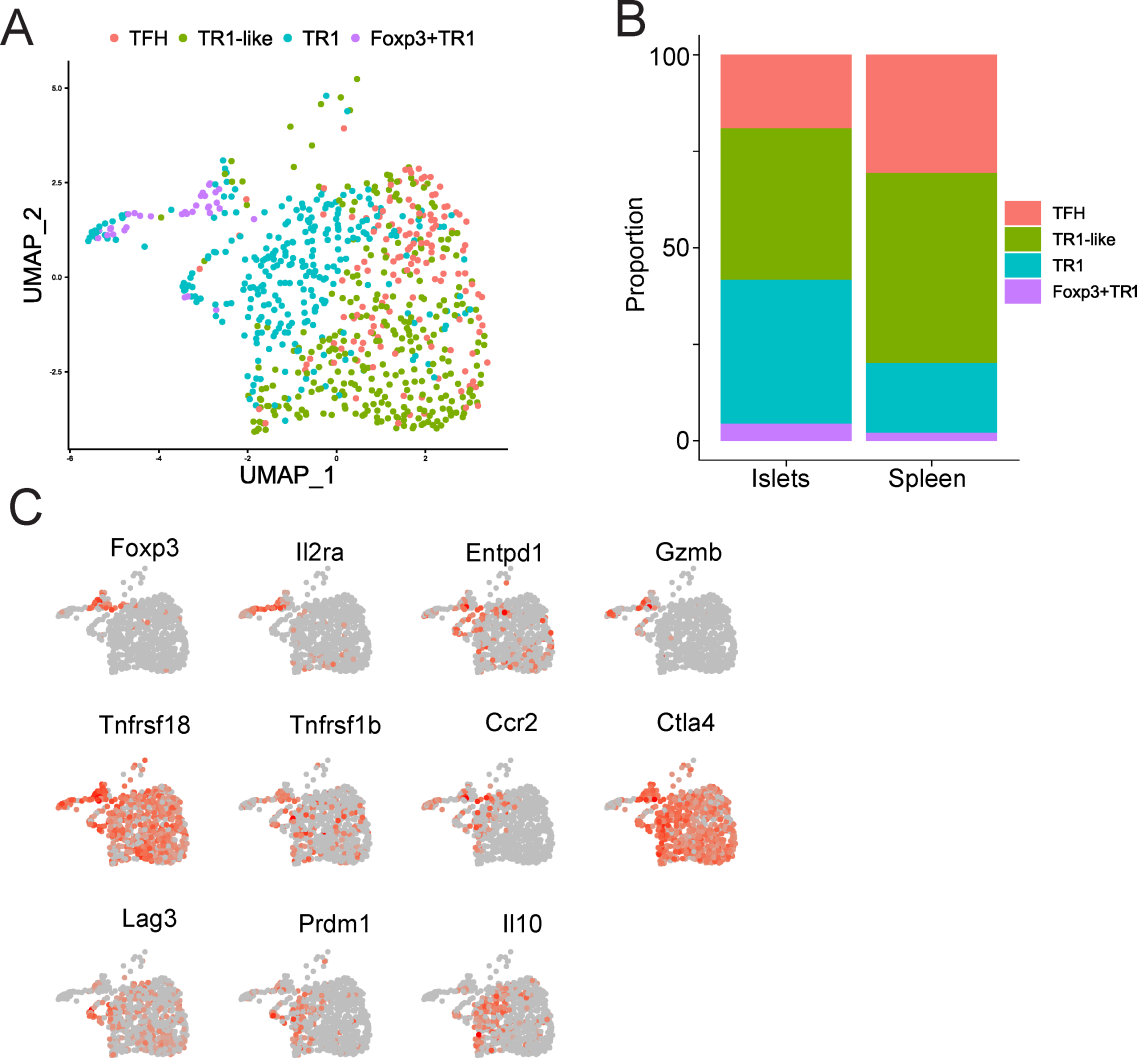
Islet-associated Foxp3^+^ TR1 cells arising in response to pMHCII-NP therapy. **(A)** UMAP scRNAseq plots for the islet-associated tetramer^+^ CD4^+^ T cells. Color shows cell type prediction based on scRNA-seq data from **Figure 1F**. **(B)** Relative distribution of TFH, TR1-like, Foxp3^–^ TR1 and Foxp3^+^ TR1 subpools within the islet- and spleen-associated tetramer^+^ cell pools of BDC2.5 mi/IA^g7^-NP-treated NOD mice, from **(A)** and **Figure 1F**, respectively (**Supplementary Data Sheet 7**). **(C)** Feature UMAP plots for representative Treg-associated genes on the UMAP reduction representation from **(A)**.

We have previously shown that the islet-associated CD4^+^ T-cells from BDC2.5mi/IA^g7^-NP-treated mice harbor significantly increased percentages of BDC2.5mi/IA^g7^ Tet^+^ T-cells than mice treated with control NPs and that these cells are enriched for the TR1 sub-cluster found in the splenic Tet^+^ T cell pool, consistent with an increased tropism for sites of inflammation (7). To ascertain whether these islet-associated TR1 cells contain Foxp3^–^ and/or Foxp3^+^ TR1 cells, we analyzed scRNAseq data from islet-associated Tet^+^ cells of BDC2.5mi/IA^g7^-NP-treated NOD mice, using the 4 cell sub-clusters found in the spleens of these mice as a reference. As shown in **Figure 3A**, both subsets were present in the islet associated BDC2.5mi/IA^g7^ Tet^+^ T cell pool. In fact, the islet-associated Tet^+^ cell pool of BDC2.5 mi/IA^g7^-NP-treated mice contained increased percentages of both Foxp3^–^ and Foxp3^+^ TR1 cells at the expense of TR1-like and TFH cells, as compared to their splenic counterparts (**Figure 3B**, **Supplementary Data Sheet 7**). The islet-derived Tet^+^ Foxp3^+^ TR1 cells displayed a gene expression profile similar to that of its splenic counterpart, including high levels of *Foxp3* and *Il2ra* (**Figure 3C**, **Supplementary Data Sheet 6**).

### pMHCII-NP-induced Foxp3^+^ and Foxp3^–^ TR1 cells share a similar open chromatin landscape

To further investigate the potential lineage relationship between Foxp3^–^ and Foxp3^+^ TR1 cells, the transitional TR1-like subset and their TFH precursors, we compared the status of their chromatin. Our previous transcriptomic and epigenetic data have suggested that TFH precursors are epigenetically poised to become TR1 cells, and that the TFH-to-TR1 conversion involves a significant contraction of the chromatin (14). We therefore compared the single-cell Multiome (scATACseq + scRNAseq) profiles of the various subsets of splenic Tet^+^ cells that arise in response to systemic BDC2.5mi/I-A^g7^-NP therapy.

**Figure 4:**
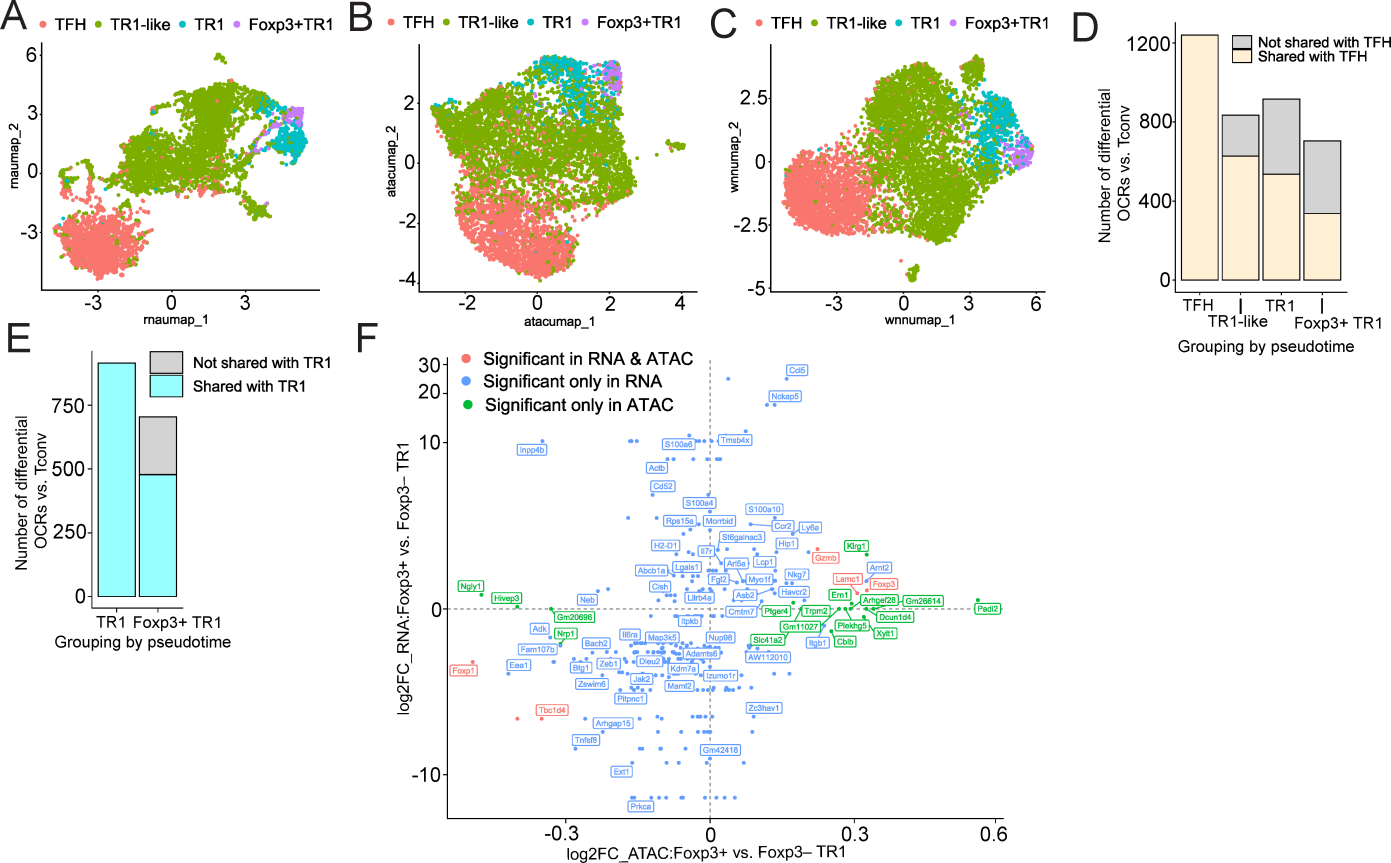
Single-cell Multiome analysis. BDC2.5mi/I-A^g7^-NP-induced Tet^+^ cell pools obtained from female NOD mice (n=4) were processed for 10X GEX^+^ATAC multiome. **(A-C)** show UMAP plots for scRNA-seq **(A)**, scATAC-seq **(B)**, and joined multidimensional analysis of scRNAseq and ATACseq data using weighted nearest neighbor (WNN) **(C)**. Color shows cell type prediction based on scRNA-seq data from **Figure 1F**. **(D, E)** Bar plots comparing the number of differential open chromatin regions between each BDC2.5mi/I-A^g7^-NP-induced Tet^+^ subpopulation relative to Tconv counterparts (adjusted P<0.05 and log2FC > 0). Color depicts open chromatin regions shared with TFH (**D**, **Supplementary Data Sheet 8**) or Foxp3^–^ TR1 (**E**, **Supplementary Data Sheet 9**). **(F)** Scatter plot of differential gene expression (y-axis) and differential chromatin accessibility (x-axis) comparing Foxp3^+^ TR1 and Foxp3^–^ TR1 cells. Only genes with either differential expression or association with a differentially accessible region are shown (adjusted P<0.05). Color depicts significance in either differential analysis. Significant in RNA & ATAC: significant in both gene expression and chromatin accessibility (adjusted P(RNA)<0.05 & adjusted P(ATAC)<0.05); Significant only in RNA: only significant at the gene expression level (adjusted P(RNA)<0.05 & adjusted P(ATAC)>=0.05); Significant only in ATAC: genes associated to chromatin regions differentially accessible between Foxp3^+^ TR1 and Foxp3^–^ TR1 cells but with no significant differential expression (adjusted P(RNA) >=0.05 & adjusted P(ATAC) <0.05) (**Supplementary Data Sheet 10**).

Mono-omic analyses of the scRNAseq and scATACseq datasets were consistent with a lineage relationship between the Foxp3^+^ TR1 cells and the other Tet^+^ T cell sub-pools. Specifically, UMAP reduction of the scRNAseq and scATACseq data, respectively, confirmed the presence of a pool of Foxp3^+^ TR1 cells having a high degree of similarity with Foxp3^–^ TR1 cells, both transcriptionally and at the level of chromatin accessibility (**Figures 4A, B**). A similar outcome was obtained when the BDC2.5mi/I-A^g7^ Tet^+^ cells were clustered using both scRNAseq and scATACSeq datasets by Weighted Nearest Neighbor (WNN) (**Figure 4C**). We next compared the chromatin status of the different BDC2.5mi/I-A^g7^ Tet^+^ cell subsets as compared to Tconv cells. Tet^+^ TFH cells showed a much higher level of chromatin accessibility than the other Tet^+^ cell subsets (1240, 834, 916 and 704 differentially open chromatin regions (OCRs) in Tet^+^ TFH, Foxp3^–^ TR1-like, Foxp3^–^ TR1 and Foxp3^+^ TR1 cells vs. Tconv cells, respectively). Most of the regions of the chromatin that remained open in the TR1 subsets were shared with Tet^+^ TFH precursors, albeit progressively less as the cells became Foxp3^+^ (OCR sharing with TFH cells: Foxp3^–^ TR1-like: 627/834 (75.2%); Foxp3^–^ TR1: 536/916 (58.5%); Foxp3^+^ TR1: 337/704 (47.9%)) (**Figure 4D**, **Supplementary Data Sheet 8**). As expected, the sharing of OCRs between Foxp3^–^ and Foxp3^+^ TR1 cells (478/704 OCRs; 67.9%) was more extensive than between either of these two cell subsets and Tet^+^ TFH cells (**Figure 4E**, **Supplementary Data Sheet 9**). Overall, this suggested that most of the chromatin accessible sites accompanying the acquisition of the TR1 cell state are already open at the Tet^+^ TFH cell stage, and that, during the TFH-to-TR1 cell conversion, TFH-specific chromatin regions are progressively closed and new chromatin regions progressively become accessible, peaking at the Foxp3^+^ TR1 cell stage.

Head-to-head comparison of the multiome-derived transcriptomes of Foxp3^+^ TR1 and Foxp3^–^ TR1 cells largely replicated the gene expression differences identified with the scRNAseq datasets (**Figure 4A**, **Supplementary Data Sheet 3**). Specifically, there were 62 differentially expressed genes between these two cell subsets (adjusted P value <0.05), 34 of which were upregulated by the former, such as *Ccl5, Ccr2, Cish, Foxp3, Gzmb, Havcr2, Ly6a, Nckap5, Neb, Nkg7, S100a6 and S100a4*, and 28 of which were downregulated, such as *Adamts6, Adk*, *Bach2*, *Il6ra, Itgb1, Izumo1r, Foxp1, Jak2, Prkca, Tbc1d4, Tnfsf8* and *Zeb1*. Differential chromatin accessibility analysis between these two subsets (scATACseq) revealed even fewer differences. Only 25 chromatin regions, associated with 22 genes, exhibited significant differential accessibility between these two cell subsets (adjusted P value <0.05). Sixteen of these regions, associated with genes such as *Foxp3, Ern1, Gzmb, Klrg1, Padi2, Trpm2* and *Xylt1*, were differentially accessible in Foxp3^+^ vs Foxp3^–^ TR1 cells. Nine other regions, associated with genes such as *Foxp1, Hivep3, Ngly1, Nrp1* and *Tbc1d4*, were differentially closed in the Foxp3^+^ TR1 subset (**Supplementary Data Sheet 10**). Notably, as shown in **Figure 4F** and **Supplementary Data Sheet 10**, chromatin accessibility differences did not significantly correlate with transcriptional differences in Foxp3^+^ vs Foxp3^–^ TR1 subsets, such that only 3 differentially upregulated genes in Foxp3^+^ vs Foxp3^–^ TR1 cells were also associated with increased chromatin accessibility (*Foxp3*, *Gzmb*, and *Lamc1*), and only 2 differentially downregulated genes in Foxp3^+^ vs Foxp3^–^ TR1 cells were associated with reduced chromatin accessibility (*Foxp1* and *Tbc1d4*).

The strong similarity between the open chromatin landscapes of Foxp3^+^ and Foxp3^–^ TR1 subsets, together with a poor correlation between changes in transcription as a function of open chromatin status (as also documented to be the case in the TFH-to-TR1 cell conversion process (14)) provided further evidence for a lineage relationship between these two cell sub-pools. These data further suggested that chromatin remodeling does not play a key role in the conversion of Foxp3^–^ TR1 cells into their Foxp3^+^ counterparts.

### Identical TCRαβ pairs in Foxp3^+^ vs. Foxp3^–^ TR1 cells

**Figure 5:**
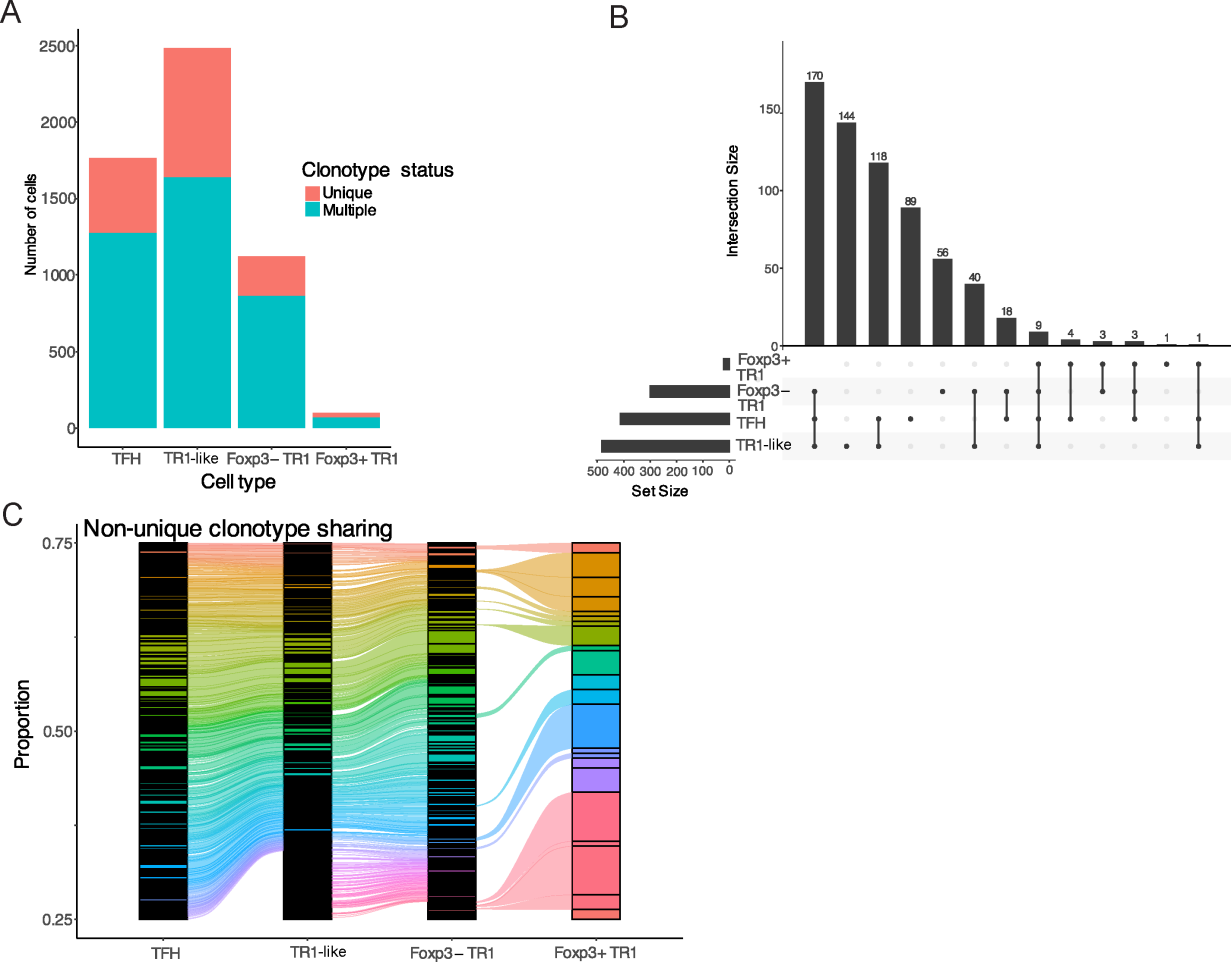
Extensive clonotype sharing among Tet^+^ TFH, TR1-like, Foxp3^–^ TR1 and Foxp3^+^ TR1 subclusters. **(A)** Absolute numbers of BDC2.5mi/IA^g7^ tetramer^+^ cells belonging to each subcluster that express unique TCRαβ sequences or TCRαβ sequences found in more than one cell. **(B)** Upset plot displaying the sharing of repeated TCRαβ pairs among the four tetramer^+^ T cell subclusters. Horizontal bars on the bottom left represent the total number of clonotypes per Tet^+^ subtype (set size). The vertical lines on the bottom right indicate the subclusters that share the TCRαβ pairs from the corresponding histogram bars (providing the total number of clonotypes for each sharing group; Intersection size). **(C)** Alluvial plot depicting the relative frequencies of all the repeated clonotypes in each Tet^+^ subcluster. Each clonotype is shown in a different color; clonotypes shared among subsets are connected by lines of the same color (**Supplementary Data Sheet 11**).

We have previously shown that the Tet^+^ cells arising in response to pMHCII-NP therapy are oligo/polyclonal and that about half of all the TCRαβ pairs that were found more than once, were present in both the Tet^+^ TFH and TR1-like/TR1 cell pools (7). Given the low frequency of Foxp3^+^ TR1 cells within the Tet^+^ T cell pool, studies of the clonotypic makeup of the Foxp3^+^ TR1 subset using Smart-Seq2 single cell sequencing technology were not informative (7). Thus, to further probe the lineage relationship between the Foxp3^+^ TR1 subset and its Foxp3^–^ counterpart, we sequenced the TCRαβ rearrangements expressed by individual Tet^+^ cells belonging to the four different cell subsets found within the BDC2.5mi/I-A^g7^ Tet^+^ T cell pool, using the 10X genomics platform. Specifically, we investigated which of the TCRαβ clonotypes found at the Foxp3^+^ TR1 cell stage were also present at previous stages of transdifferentiation along the TFH-TR1 cell axis. We obtained TCR sequences for 5,474 of the 7,037 cells that were scRNA-sequenced (77%). These cells could be classified into 1,628 clonotypes, of which 972 (59.7%) were unique (i.e., not repeated). These unique clonotypes were distributed evenly across the four different Tet^+^ cell subclusters (TFH: 492 (27.8%); Foxp3^–^ TR1-like: 847 (34%); Foxp3^–^ TR1: 259 (23%); and Foxp3^+^ TR1: 30 (30%)) (**Figure 5A**). The Tet^+^ Foxp3^+^ TR1 subset expressed a total of 21 TCRαβ pairs found in more than one cell across the entire dataset. Remarkably, 20 of these TCRαβ pairs (95.2%) were found in at least one of the other three Tet^+^ T cell subsets, 15 (71.4%) were found in the Foxp3^–^ TR1-like subset (**Figure 5C**), and 9 (45%) were shared by all four subsets (**Figures 5B, C**) (**Supplementary Data Sheet 11**). These data further supported our contention that, like their Foxp3^–^ counterparts, the Foxp3^+^ TR1 cells arise from TFH precursors.

### Development of the Foxp3^+^ TR1 subset is preceded by cognate TFH cell expansion and requires BLIMP-1 and IRF4

The TFH-to-TR1 cell conversion and therapeutic activity of pMHCII-NPs requires the expression of BLIMP-1 (encoded by *Prdm1*) (7); treatment of NOD.*Cd4-Cre*.*Prdm1^loxP/loxP^* mice with BDC2.5mi/I-A^g7^-NP triggered the expansion of cognate TFH cells and the formation of a reduced population of transitional TR1-like cells but could not generate fully differentiated Foxp3^–^ TR1 cells.

**Figure 6:**
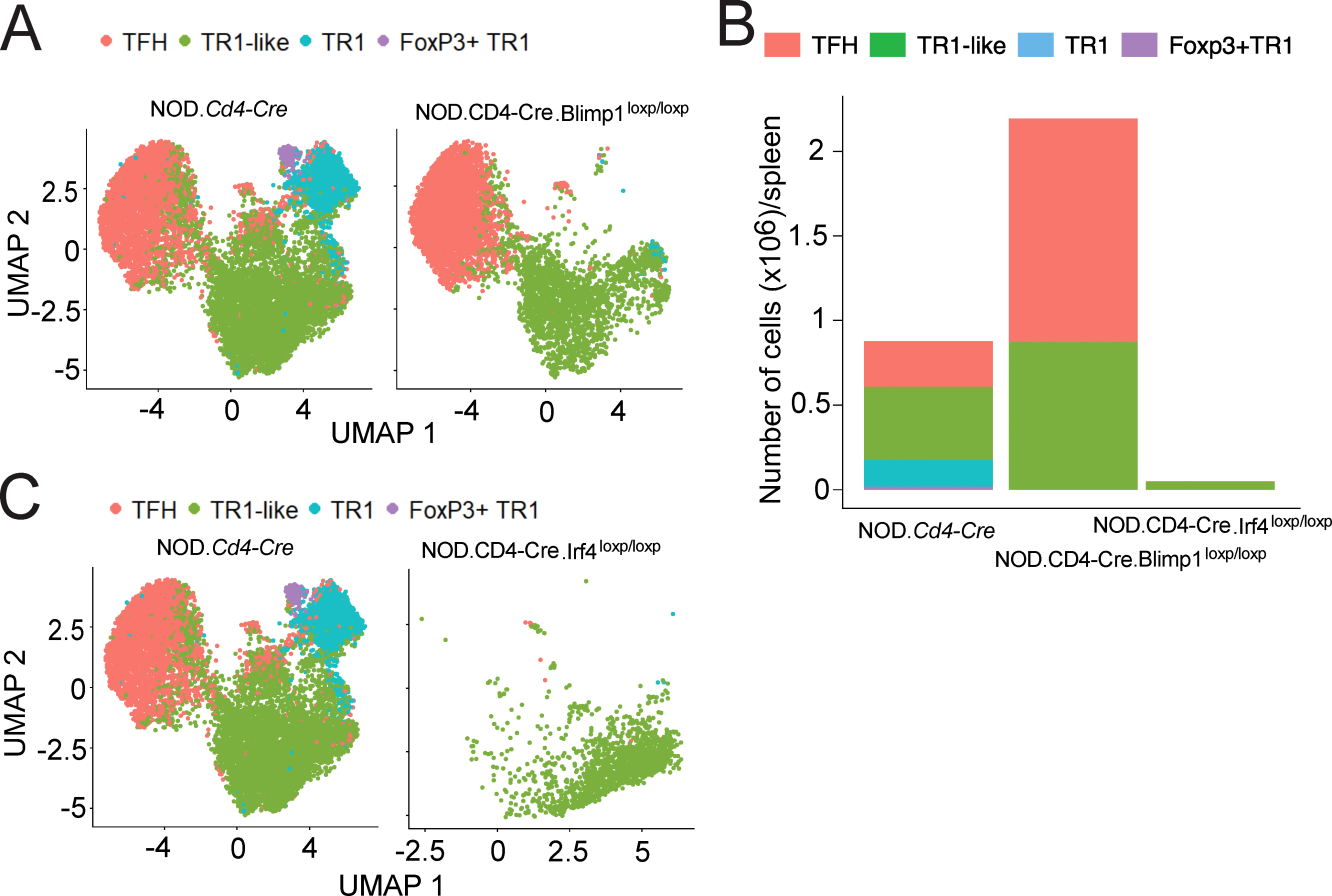
Absence of Foxp3^+^ TR1 cells in pMHCII-NP-treated mice unable to generate Foxp3^–^ TR1 or TFH cells. **(A)** UMAP plots of 10x Genomics scRNAseq data for sorted BDC2.5 mi/IA^g7^ tetramer^+^ (Tet^+^) cells from BDC2.5 mi/IA^g7^-NP-treated NOD.*Cd4-Cre.Prdm1^loxP/loxP^* mice (*n* = 2) vs. NOD.*Cd4-Cre* (n=3). **(B)** Fraction of total cells corresponding to each Tet^+^ subcluster from NOD.*Cd4-Cre*, NOD.*Cd4-Cre.Prdm1^loxP/loxP^* and NOD.*Cd4-Cre.Irf4^loxP/loxP^* mice. **(C)** UMAP plots for scRNAseq data for NOD.*Cd4-Cre.Irf4^loxP/loxP^* (*n* = 2) vs. NOD.*Cd4-Cre* mice (n=3). Cell subsets were identified via prediction annotation using the 10x Genomics scRNAseq data from **Figures 1B, F** (wildtype control dataset) as a reference.

Analysis of scRNAseq data for the Tet^+^ cells of BDC2.5mi/I-A^g7^-NP-treated NOD.*Cd4-Cre*.*Prdm1^loxP/loxP^* mice revealed that pMHCII-NP therapy failed to generate not only the Foxp3^–^ TR1 subset, but also its Foxp3^+^ counterpart (**Figures 6A, B**). We note that this is in stark contrast to the dispensable role that BLIMP-1 plays in the development of conventional Foxp3^+^CD25^+^ Treg and TFR cells (8); in fact, NOD.*Cd4-Cre*.*Prdm1^loxP/loxP^* mice harbor increased percentages and absolute numbers of Foxp3^+^CD25^+^ Treg and TFR cells as compared to NOD.*Cd4-Cre* mice (7).

The above results were further substantiated by studying BDC2.5mi/I-A^g7^-NP-treated NOD.*Cd4-Cre*.*Irf4^loxP/loxP^* mice, in which pMHCII-NP therapy can barely expand the tetramer^+^ CD4^+^ T cell pool (**Figure 6B**). These mice harbored much smaller pools of cognate Tet^+^ cells than NOD.*Cd4-Cre* controls (**Figure 6B**), and such pools were exclusively composed of Foxp3^–^ TR1-like cells, therefore lacking TFH cells, Foxp3^–^ TR1 and Foxp3^+^ TR1 cells (**Figures 6B, C**). This outcome suggests a dual role for IRF4 in the TFH-to-TR1 transdifferentiation pathway: as a necessary transcription factor for TFH cell genesis/homeostasis (sustaining the TFH cell state and inhibiting TR1-like cell formation), and as a catalyst for the pMHCII-NP-induced differentiation of TR1-like cells into terminally differentiated Foxp3^–^ and Foxp3^+^ TR1 cells.

### pMHCII-NP-induced formation of Foxp3^+^ TR1 cells in TFH cell-transfused hosts

**Figure 7:**
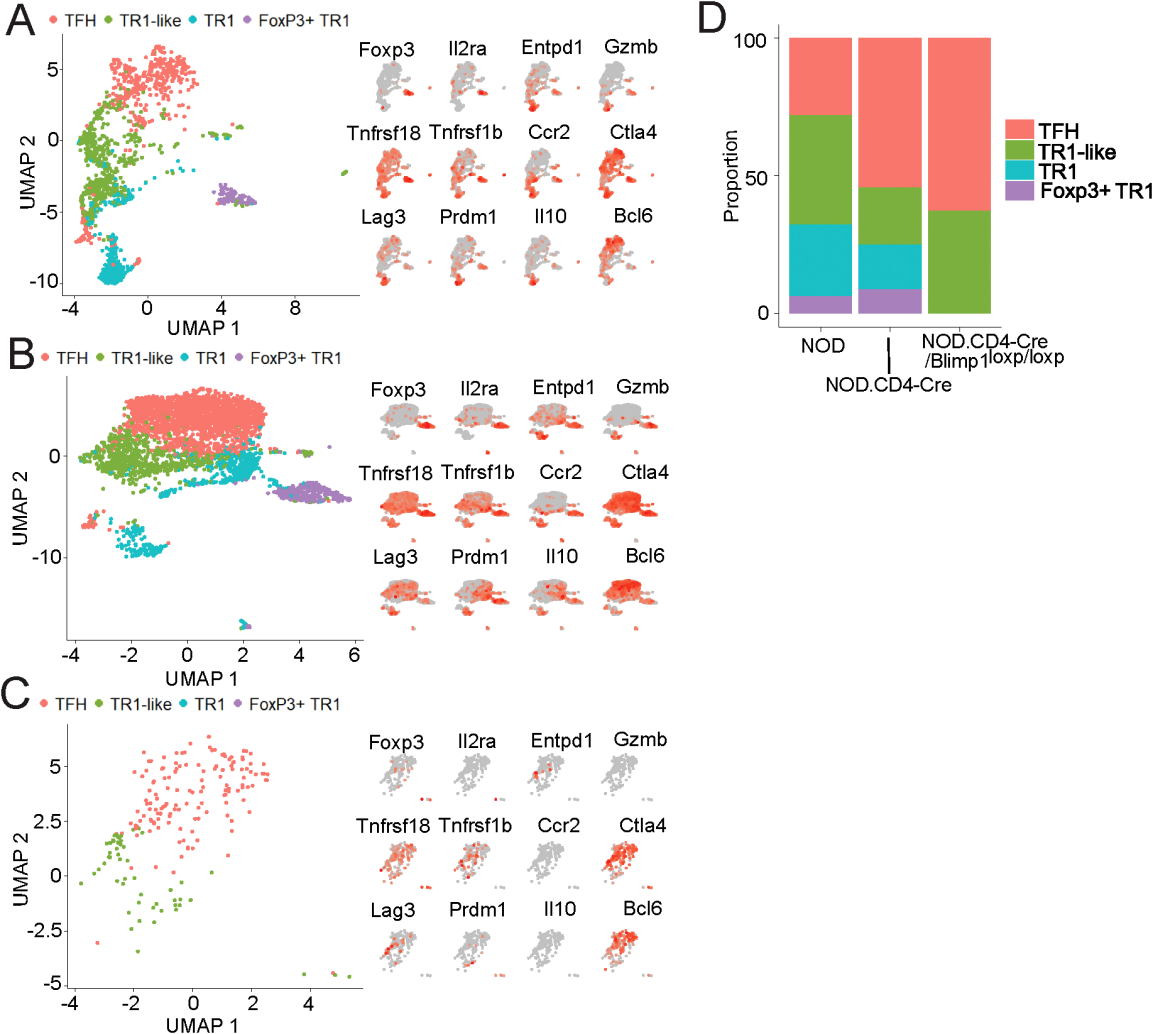
pMHCII-NP-induced formation of terminally differentiated TR1 cells from PD-1^hi^CXCR5^hi^ precursors. 10x Genomics scRNAseq data for sorted BDC2.5 mi/IA^g7^ Tet^+^ cells from BDC2.5 mi/IA^g7^-NP-treated NOD.*Scid* mice adoptively transferred with PD-1^hi^CXCR5^hi^ (TFH) cells from different donors. **(A-C)** UMAP plots with pMHCII-NP-induced Tet^+^ subpopulations arising from TFH cells from wild-type NOD **(A)**, NOD.*Cd4-Cre* mice **(B)** and NOD.*Cd4-Cre.Prdm1^loxP/loxP^* mice **(C)** (left panels) and feature plots for representative Treg markers gene transcripts (right panels). The data correspond to cells pooled from 2 hosts for each treatment group and donor strain. **(D)** Relative percentages of pMHCII-NP-induced Tet^+^ subpopulations within the Tet^+^ cell pools of each donor/host combination.

BDC2.5mi/I-A^g7^-NPs can trigger cognate TR1-like and TR1 formation in NOD.*Scid* hosts engrafted with FACS-sorted splenic CXCR5^hi^PD-1^hi^CD4^+^ T-cells (TFH cells) (7). As predicted by the data described herein, analyses of the corresponding datasets using the Foxp3^+^ TR1-specific transcriptome as a reference (**Figure 1F**) demonstrate that the Tet^+^ CD4^+^ T cells arising in these mice in response to BDC2.5mi/I-A^g7^-NPs also contain a Foxp3^+^ subset (**Figures 7A, D**). Furthermore, whereas the Tet^+^ cells arising in BDC2.5mi/I-A^g7^-NP-treated NOD.*Scid* hosts transfused with TFH cells from NOD.*Cd4-Cre* donors (control strain) harbored the 4 Tet^+^ subsets found in wild-type mice (**Figures 7B, D**), the Tet^+^ cells arising BDC2.5mi/I-A^g7^-NP-treated NOD.*Scid* mice transferred with TFH cells from NOD.*Cd4-Cre*.*Prdm1^loxP/loxP^* donors (unable to generate Foxp3^–^ and Foxp3^+^ TR1 cells; see **Figure 6**) harbored neither Foxp3^–^ TR1 nor Foxp3^+^ TR1 cells (**Figures 7C, D**). These data thus provided additional compelling evidence for a TFH cell origin of the Foxp3^+^ TR1 cell subset.

### The Foxp3^+^ TR1 cell subset contributes to the therapeutic activity of pMHCII-NPs

We next sought to ascertain whether the Foxp3^+^ TR1 cells arising in response to pMHCII-NP therapy have immunoregulatory properties *in vivo*. We focused these studies on the Myelin Oligodendrocyte Glycoprotein peptide (pMOG_35-55_)-induced model of experimental autoimmune encephalomyelitis (EAE) in B6 mice, where pMOG_38-49_/I-A^b^-NP therapy can reverse established disease by inducing the formation and expansion of cognate TR1 cells (7, 10). Unlike NOD mice, where disease occurs spontaneously, development of EAE requires immunization of the mice with pMOG_35-55_ in the presence of Complete Freund’s Adjuvant (CFA) and the administration of Pertussis toxin. As a result, both pMHCII-NP-treated and untreated EAE mice harbor pMOG_38-49_/IA^b^ Tet^+^ CD4^+^ T-cells, albeit at significantly different frequencies (7). Therefore, to minimize the presence of contaminating, immunization-induced Tet^+^ cells in the cell isolates used for scRNAseq, we focused our scRNAseq experiments on Tet^+^ cells contained within the CD4^+^ICOS^+^PD-1^+^ gate (**Supplementary Figure 5**); both ICOS and PD-1 are expressed by the cognate TFH and TR1 cells that arise in response to pMHCII-NP therapy (7, 13).

**Figure 8:**
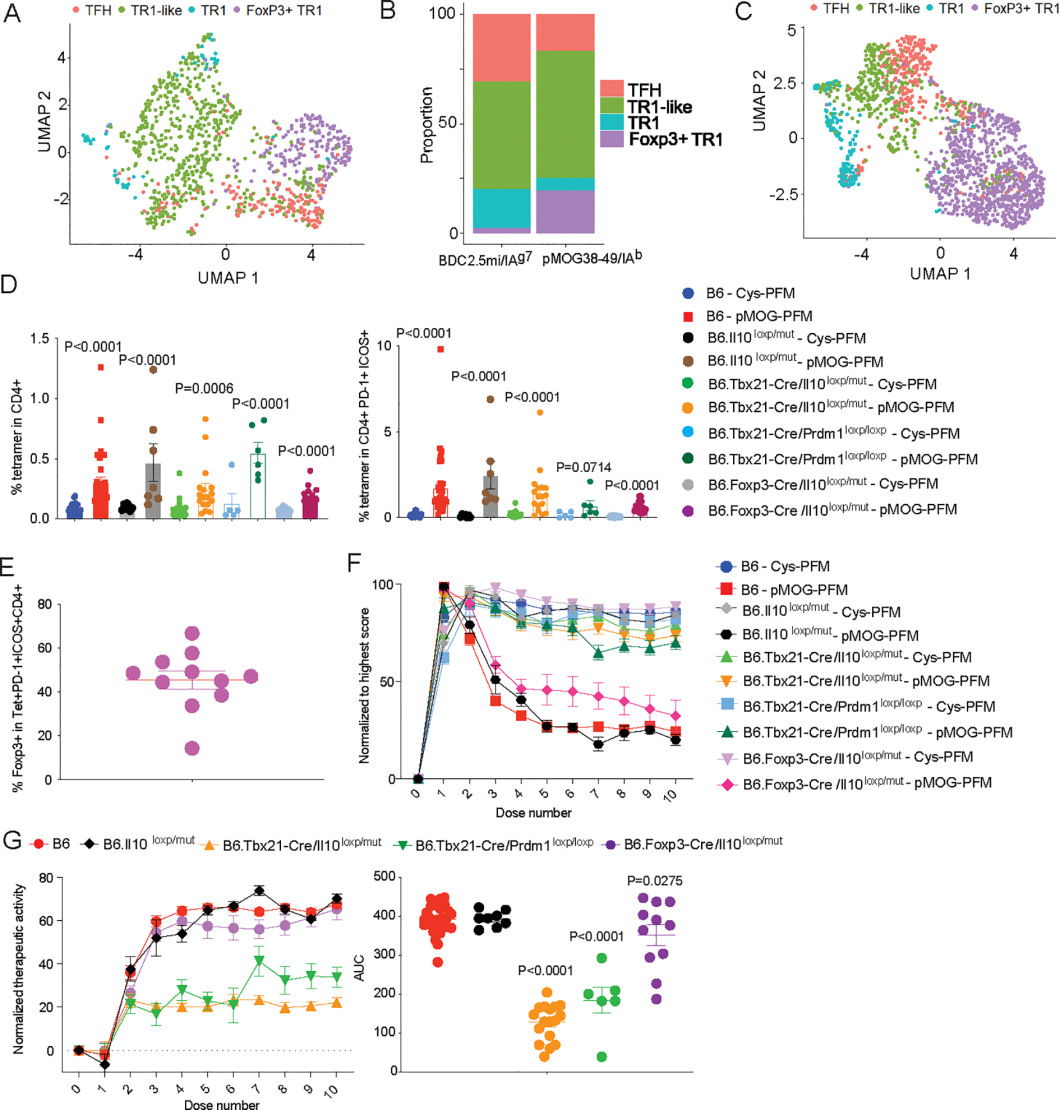
Therapeutic vs. pharmacodynamic activity of pMHCII-NP therapy in mice unable to express *Il10* or *Prdm1* in Foxp3^–^TR1 cells or Foxp3^+^ cells. **(A)** UMAP visualization of scRNAseq data corresponding to pMOG_38–49_/IA^b^-NP-induced splenic tetramer^+^ cells from B6 mice with EAE, sorted as described in **Supplementary Figure 5**. Cell type identity (TFH, TR1-like, Foxp3^–^ TR1 and Foxp3^+^ TR1) was annotated using the **Figure 1F** dataset. **(B)** Relative proportions of pMHCII-NP-induced Tet^+^ subpopulations cells within the Tet^+^ cells for BDC2.5mi/I-A^g7^-NP and pMOG_38–49_/IA^b^–NP treated mice, respectively. **(C)** UMAP visualization of scRNAseq data corresponding to pMOG_38–49_/IA^b^-NP-induced splenic Tet^+^ cells from B6.*Tbx21-Cre.Il10^loxP/mut^* mice with EAE, sorted as described in **Supplementary Figure 5**. Cell type identity (TFH, TR1-like, Foxp3^–^ TR1 and Foxp3^+^ TR1) was annotated using the **Figure 1F** dataset. **(D)** Percentages of splenic pMOG_38-49_/I-A^b^ Tet^+^ cells in the CD4^+^ pool (left) or CD4^+^ICOS^+^PD-1^+^ pool (right) in B6, B6.*Il10^loxP/mut^*, B6.*Tbx21-Cre*.*Il10^loxP/mut^*, B6.*Tbx21-Cre*.*Prdm1^loxP/loxP^* and B6.*Foxp3^-^Cre*.*Il10^loxP/mut^* mice (both males and females) upon pMOG_38-49_/I-A^b^-NP (n=28, 7, 17, 6 and 11, respectively) vs. Cys-NP treatment (n=23, 7, 17, 5 and 9, respectively) (10 doses over 5 weeks, starting when the mice reached a score >1.5/5). Data were from 6, 1, 4, 1 and 1 experiments, respectively. **(E)** Percentage of eYFP (Foxp3^+^) cells within the CD4^+^ICOS^+^PD-1^+^ pool of B6.*Foxp3^-^Cre*.*Il10^loxP/mut^* mice. **(F)** Normalized EAE scores upon pMOG_38-49_/I-A^b^-NP or Cys-NP treatment from the mice in **(D)** (pMHCII-NP-treated: n=28, 8, 17, 7 and 11, respectively; Cys-NP-treated: n=28, 9, 17, 5 and 9, respectively) from 1-6 experiments per strain and NP-type. **(G)** Left: plots of normalized therapeutic activity generated by subtracting the average EAE scores of Cys-NP-treated mice at each individual dose from the individual mouse EAE scores corresponding to pMHCII-NP-treated mice. The lower the value, the greater the therapeutic activity. Right: area under the curve (AUC) values (doses 4-10) for the individual mouse plots used to generate the left panel (n=28, 8, 17, 7 and 11, from left to right). Data correspond to average ± SE of the mean and were compared to B6 via one-way ANOVA.

We first asked whether pMOG_38-49_/I-A^b^-NP therapy in this disease model and genetic background can also elicit the formation of the Foxp3^+^ TR1 cell subset. Indeed, the Tet^+^ ICOS^+^PD-1^+^CD4^+^ T cells isolated from pMOG_38–49_/IA^b^-NP treated mice harbored the four antigen-specific T cell subsets described in BDC2.5mi/I-A^g7^-NP-treated NOD mice (**Figure 8A**), albeit with different frequencies. That is, the pMOG_38–49_/IA^b^ tetramer^+^ pool contained smaller percentages of TFH cells and Foxp3^–^ TR1 cells at the expense of increased percentages of Foxp3^–^ TR1-like and Foxp3^+^ TR1 cells (**Figure 8B**). A similar outcome was obtained in pMOG_38–49_/IA^b^-NP-treated B6.*Tbx21-Cre.Il10^loxP/mut^* mice (**Figure 8C**), in which TR1 cells lack *Il10* and pMHCII-NP therapy fails to induce disease reversal (7). Thus, whereas IL-10 expression is dispensable for pMHCII-NP-induced Foxp3^–^ and Foxp3^+^ TR1 cell formation, it is required for therapeutic activity.

Having established that pMOG_38-49_/I-A^b^-NP therapy triggers the formation of a cognate Foxp3^+^ TR1 cell pool in B6 mice with EAE, we next set out to investigate its contribution to the therapeutic activity of this compound in this model. Since expression of *Il10* specifically occurs at both the Foxp3^–^ and Foxp3^+^ TR1 cell stages, and IL-10 is necessary for therapeutic activity in all the animal models of autoimmunity tested to date, including EAE (7, 10), we studied the pharmacodynamic and therapeutic effects of pMOG_38-49_/I-A^b^-NP therapy in pMOG_35-55_-immunized B6.*Foxp3^-^Cre*.*Il10^loxP/mut^* mice, to delete *Il10* at the Foxp3-expressing TR1 cell stage. We compared these effects to those obtained in: (1) wild-type B6 and B6.*Il10^loxP/mut^* mice, as controls; (2) B6.*Tbx21-Cre*.*Il10^loxP/mut^* mice, to delete *Il10* at the *Tbx21*-expressing TR1-like stage, which precedes Foxp3^–^ TR1 and Foxp3^+^ TR1 formation; and (3) B6.*Tbx21-Cre*.*Prdm1^loxP/loxP^* mice, to blunt the development of Foxp3^–^ and Foxp3^+^ TR1 cells from their Foxp3^–^ TR1-like precursors (see further above). Although *Tbx21* can also be expressed by encephalitogenic T cells, B6.*Tbx21-Cre.Il10^loxP/mut^* and B6.*Tbx21-Cre*.*Prdm1^loxP/loxP^* mice treated with control Cys-coated NPs, hence unable to generate antigen-specific TR1 cells, displayed EAE scores similar to those seen in wildtype B6 mice. Thus, absence of *Il10* or *Prdm1* in *Tbx21*-expressing CD4^+^ T cell subsets does not significantly alter disease onset or progression.

pMOG_38-49_/IA^b^-NP therapy elicited similar pharmacodynamic activity (i.e. expansion of cognate Tet^+^ CD4^+^ and Tet^+^ ICOS^+^PD-1^+^CD4^+^ T cells) in B6.*Foxp3^-^Cre*.*Il10^loxP/mut^* mice as compared to B6, B6.*Il10^loxP/mut^*, B6.*Tbx21-Cre*.*Il10^loxP/mut^* and B6.*Tbx21-Cre*.*Prdm1^loxP/loxP^* mice (**Figure 8D**). Over 40% of the Tet^+^ ICOS^+^PD-1^+^CD4^+^ T cells of the pMOG_38-49_/IA^b^-NP-treated B6.*Foxp3^-^Cre*.*Il10^loxP/mut^* mice expressed Foxp3 promoter-driven eGFP (**Figure 8E**).

As shown in **Figure 8F**, showing EAE scores normalized to the highest score in each strain, pMOG_38-49_/IA^b^-NP therapy had a slightly impaired therapeutic activity in B6.*Foxp3^-^Cre*.*Il10^loxP/mut^* mice as compared to B6 and B6.*Il10^loxP/mut^* mice. Raw EAE scores as a function of days after immunization, and EAE scores synchronized to the beginning of treatment along with the corresponding areas under the curve (AUC) are shown on **Supplementary Figures 6A, B**, respectively. **Supplementary Figure 6C** correspond to EAE scores and AUCs normalized to the scores at beginning of treatment (as opposed to the highest score in each strain in **Figure 8F**). To more accurately compare the therapeutic data, we subtracted the EAE scores of each pMHCII-NP-treated cohort from its Cys-NP-treated counterpart (from **Figure 8F**), to generate a single normalized curve for each strain (**Figure 8G**, left). Calculation of the AUC for each strain revealed that deletion of *Il10* in the Foxp3^+^ T cells of B6.*Foxp3^-^Cre*.*Il10^loxP/mut^* mice significantly impaired the therapeutic activity of pMHCII-NPs (**Figure 8G**, right). Direct comparison of the AUC values corresponding to the raw EAE scoring data synchronized to the initiation of treatment (**Supplementary Figure 6B**) showed statistically significant differences between pMHCII-NP (but not Cys-NP) treated wild-type B6 and B6.*Foxp3^-^Cre*.*Il10^loxP/mut^* mice (P=0.0437), indicating that this effect was not an artefact of normalization. As expected, deletion of *Il10* or *Prdm1* at the transitional TR1-like stage (hence also in both Foxp3^–^ and Foxp3^+^ TR1 cells) impaired the therapeutic activity of the pMHCII-NP compound even further (**Figure 8G**). Similar results were obtained when therapeutic activity curves and AUC values were generated using EAE scores normalized to the scores at the beginning of treatment, as opposed to the highest scores in each strain (**Supplementary Figure 6D**). Thus, pMHCII-NPs possess reduced therapeutic activity in mice in which the pMHCII-NP-induced Tet^+^ Foxp3^+^ TR1 subset cannot produce IL-10, and almost no therapeutic activity in mice in which neither the Foxp3^+^ nor the Foxp3^–^ TR1 subsets can produce IL-10, or in mice that lack the Blimp-1-dependent, terminally differentiated Foxp3^–^ and Foxp3^+^ TR1 sub-pools.

## Discussion

We have recently shown that systemic delivery of autoimmune disease-relevant pMHCII-NPs expands cognate TFH cells and triggers the transdifferentiation of these TFH cells into disease-suppressing Foxp3^–^ TR1-like cells (7, 9–13). Here, we show that differentiation of TFH cells into Foxp3^–^ TR1 cells also involves the generation of a novel Foxp3^-^expressing TR1 cell subset that increases in size upon treatment withdrawal and is different than other peripheral Foxp3^+^ Treg cell subsets, including TFR cells. We further demonstrate that these pMHCII-NP-induced Foxp3^+^ TR1 cells, like their Foxp3^–^ counterparts (7, 13), arise from TFH precursors in a BLIMP-1 and IRF4-dependent manner, unlike the case for conventional Foxp3^+^ Treg cells. In addition, pMHCII-NPs can trigger Foxp3^+^ TR1 cell formation in NOD.*Scid* hosts engrafted with purified TFH cells from wild-type mice, but not in hosts engrafted with purified TFH cells from mice lacking BLIMP-1 in T cells. Thus, TFH cells can transdifferentiate into both Foxp3^–^ and Foxp3^+^ TR1 cells.

Detailed transcriptomic analyses of the small cluster of Foxp3^+^ T cells contained within the pMHCII-NP-induced Tet^+^ pool 2-3 days after the last dose indicated that these cells were highly related to the previously described Tet^+^ TR1 cell subset. In addition to expressing high levels of *Foxp3*, these cells had further upregulated or downregulated genes whose expression progressively changed as TFH cells became TR1-like, Foxp3^–^ TR1 and, ultimately, Foxp3^+^ TR1, such as *Ccr2, Ctla4, Entpd1, Gzmb, Il2ra*, *Il10*, *Lag3*, *Prdm1, Tnfrsf18* and *Tnfrsf1b*. Most of the TFH genes that were downregulated by the Foxp3^–^ TR1 subset as it evolved from the transitional TR1-like sub-pool, remained downregulated at the Foxp3^+^ TR1 stage, and most of the genes that were upregulated by Foxp3^–^ TR1 cells as compared to Tet^+^ TFH (and the transitional Tet^+^ Foxp3^–^ TR1-like) cells, including *Ccr5, Ctla4, Lag3, Il10, and Prdm1*, remained upregulated at the Foxp3^+^ TR1 stage. We acknowledge that our data strongly suggest, but do not demonstrate, that Foxp3^+^ TR1 cells arise directly from Foxp3^–^ TR1 cells rather than independently from TFH cells. Specifically, our trajectory analyses suggest a closer relationship between Foxp3^–^ TR1 and Foxp3^+^ TR1 cells than between Foxp3^+^ TR1 cells and TFH cells (**Figure 1G**). In addition, the transcriptional and chromatin accessibility profiles of these two subsets are more similar to each other than to those corresponding to TFH cells (**Figure 4**). Significant sharing of clonotypic TCRαβ pairs (equivalent to cell-specific “barcode” labelling) between the Foxp3^–^ and Foxp3^+^ TR1 subsets, similar in extent to that described for the TFH-to-Foxp3^–^ TR1 comparison (7, 13), provides further evidence for a direct Foxp3^–^ TR1-to-Foxp3^+^ TR1 cell conversion. Specifically, 71.4% of the clonotypes found in the Foxp3^+^ TR1 subset were also present in the TR1-like and Foxp3^–^ TR1 subsets, while only 15% of Foxp3^+^ TR1 clonotypes were uniquely shared with TFH cells (**Figure 5**). This pattern suggests a stronger clonal connection between Foxp3^+^ and Foxp3^–^ TR1 subsets. Notwithstanding these observations, demonstration of a direct Foxp3^–^ to Foxp3^+^ TR1 conversion will require additional experimentation.

The Foxp3^+^ TR1 subset became particularly apparent in studies of the splenic pMHCII-NP-induced Tet^+^ CD4^+^ T cell subset upon treatment withdrawal. There was a progressive enrichment of this subset in the spleen-resident Tet^+^ CD4^+^ T cell pool persisting 5-10 weeks after the cessation of treatment, in association with a progressive reduction in the size of the Foxp3^–^ TR1-like/TR1 cell pool. Pseudotime trajectory analysis of all the subsets identified in these experiments, including those isolated 5 and 10 weeks post-treatment withdrawal, generated a pathway that involves progressive differentiation of TFH cells into Foxp3^–^ TR1-like and TR1 cells, and ultimately, Foxp3^+^ TR1 cells. Although it is unclear why treatment withdrawal would promote the conversion of local Foxp3^–^ TR1 cells into their the Foxp3^+^ TR1 counterparts, it is possible that this cellular conversion is suppressed by the sustained and profound TCR ligation afforded by pMHCII-NPs. In fact, in support of this possibility, there was a general downregulation of subset-specific gene expression in all the pMHCII-NP-induced T cell subsets post-treatment withdrawal, including the Foxp3^+^ TR1 subset. Likewise, the Tet^+^ TFH cells appeared to acquire a memory TFH-like profile (15) post-treatment withdrawal. Alternatively, some of these changes in the subset composition of the splenic Tet^+^ T cell pool may be due to differences in the rates with which specific subsets (e.g. the Foxp3^–^ TR1) migrate out of the spleen into the bloodstream in search for sites of inflammation. We further entertain the possibility that the Foxp3^–^ and Foxp3^+^ TR1 subsets arising during treatment are programmed to rapidly exit the spleen, and that only the Foxp3^+^ TR1 cells that downregulate specific homing receptors post-treatment withdrawal remain in the spleen.

We have recently shown that pMHCII-NP-induced TFH cells undergo massive closure of open chromatin regions as they transdifferentiate into Foxp3^–^ TR1-like and TR1 cells (14). Furthermore, most of the open chromatin regions that remain open in the TR1 subset and, especially, the transitional TR1-like subset were already open at the TFH cell stage (14). Additional analyses of the scMultiome-derived scRNAseq data confirmed that chromatin closure during the TFH to Foxp3^–^ TR1 cell conversion is accompanied by an equally extensive downregulation of gene expression (14). Here, in agreement with the proposed lineage relationship between TFH, Foxp3^–^ TR1 and Foxp3^+^ TR1 cells, we find that a large fraction of the chromatin accessible sites found at the Foxp3^+^ TR1 cell stage are already open at the Foxp3^–^ TR1 cell stage, and that transcriptomic changes during this cellular transition cannot be accounted for by changes in accessible chromatin. Together with the other data described herein, these observations further support the proposed lineage relationship between these two cell sub-pools.

Comparison of the transcriptional profile of the pMHCII-NP-induced Foxp3^+^ TR1 subset to other murine Foxp3^+^ Treg cell subsets isolated from skin, colon, and lymphoid tissue (including several lymph nodes and spleen) (18) revealed clearly distinct transcriptional signatures. Importantly, the Tet^+^ Foxp3^+^ TR1 subset expresses higher levels of numerous Foxp3^+^ Treg cell-associated genes, including *Ctla4, Foxp3, Gzmb, Il10, Lag3* and *Prdm1*. The Foxp3^+^ TR1 subset displays a transcription factor expression profile that is remarkably similar to that seen in Foxp3^–^ TR1 cells yet remarkably different than that seen in conventional Foxp3^+^ Treg subsets. The pMHCII-NP-induced Foxp3^+^ TR1 subset also expresses significantly higher levels of genes involved in cell migration and trafficking as compared to conventional splenic Treg cells, suggesting that the Tet^+^ Foxp3^+^ TR1 subset is programmed to migrate to extra-lymphoid sites and participate in the recruitment of additional Foxp3^–^ and Foxp3^+^ TR1 cells, presumably to effect immunoregulation. Indeed, transcriptional analyses of the islet-associated tetramer^+^ CD4^+^ T cells in mice treated with BDC2.5mi/IA^g7^-NPs confirmed the presence of both the Foxp3^–^ and Foxp3^+^ TR1 subsets. Notwithstanding the unique transcriptional properties of the splenic pMHCII-NP-induced Foxp3^+^ TR1 subset, gene expression profiles can undergo significant changes in response to environmental cues, such as cytokines, tissue type or disease states. Therefore, it is likely that the transcriptional make-up of this subset will change as a function of these cues. It is also worth noting that this study only reports on the transcriptional profiles of the Foxp3^–^ and Foxp3^+^ TR1 Tet^+^ T cell pools post treatment withdrawal. Since the TFH-TR1 transdifferentiation pathway involves dynamic changes in gene expression leading to cell conversion events, it will be interesting to enumerate the presence of these T cell subsets as a function of dose number. Our previous studies of mice treated with 0, 1, 3, 5, 7 and 10 doses of pMHCII-NPs showed that treatment triggered progressive increases in both the absolute numbers of pMHCII-NP-induced Tet^+^ TFH- and TR1-like cells and in the Tet^+^ TR1:TFH-like cell ratios as a function of dose number, but these studies used mass cytometry rather than scRNA-seq and the antibody panel used could not distinguish between the Foxp3^–^ and Foxp3^+^ TR1 subsets.

The different lineage origin of Foxp3^+^ TR1 vs conventional Foxp3^+^ Treg cells suggested by the above transcriptional differences is further supported by clearly different roles of specific transcription factors other than Foxp3 in the development of these T cell subsets. T cell-specific deletion of *Prdm1* (encoding BLIMP-1, a zinc-finger motif-containing transcriptional repressor that antagonizes Bcl-6 expression and function in both B- and T-cells, including TFH cells (19–22)), abrogated the ability of pMHCII-NPs to generate both Foxp3^–^ and Foxp3^+^ TR1 cells, without impairing the preceding TFH cell expansion step. We note that this is in stark contrast to the dispensable role that BLIMP-1 plays in the development of conventional Foxp3^+^ Treg cells; in fact, mice carrying a T cell specific deletion of *Prdm1* harbor increased percentages and absolute numbers of Foxp3^+^CD25^+^ Treg and TFH cells (~3 and ~4-fold, respectively) as compared to wild-type mice (7). Thus, although the role of BLIMP-1 in the genesis of Foxp3^+^ TR1 cells from Foxp3^–^ TR1 precursors remains to be determined, our data indicate that BLIMP-1 is absolutely required for generation of the latter (7). The lineage relationship between Foxp3^–^ and Foxp3^+^ TR1 cells elicited by pMHCII-based nanomedicines was further supported by the absence of these two TR1 cell pools in mice carrying IRF4-deficient T cells. Of note, and unlike BLIMP-1, IRF4 plays a key role in the development/homeostasis of effector Foxp3^+^ Treg cells (in addition to TFH cells) (23).

Our work further shows that TFH cells lie upstream of BLIMP-1, and upstream of both Foxp3^–^ and Foxp3^+^ TR1 cells in pMHCII-NP-induced TR1 cell formation. These observations were confirmed by investigating the ability of pMHCII-NPs to trigger Foxp3^–^ and Foxp3^+^ TR1 cell formation in NOD.*Scid* hosts engrafted with splenic TFH cells from wild-type mice or mice carrying a T cell-specific deletion of *Prdm1*. Whereas these compounds readily triggered the formation of all four cognate T cell subsets in hosts carrying wild-type TFH cells, they were completely unable to trigger Foxp3^–^ and Foxp3^+^ TR1 cell formation in hosts carrying TFH cells from *Prdm1*-deficient donors. It is noteworthy that a significant number of genes whose expression has been previously associated with BLIMP-1 expression, including *Il10, Ctla4, Lag3, Icos, Havcr2, Tnfrsf4* and *Tnfrsf18*, among others (24) are progressively upregulated during the TFH-to-TR1 cell conversion to reach peak levels in Foxp3^+^ TR1 cells.

Adoptive transfer experiments using purified Tet^+^ Foxp3^–^ TR1 cells rather than TFH cells are not technically feasible/practical because fewer than 20,000 Tet^+^ Foxp3^–^ TR1 cells can be purified from a single treated mouse. Furthermore, given the strong phenotypic similarities between Foxp3^–^ and Foxp3^+^ TR1 subsets, it is not possible to purify one from the other using flow cytometry without the use of a nuclear stain for Foxp3, which would preclude post-isolation cell survival. Furthermore, since these cells do not proliferate, it would be extremely difficult to track the conversion of even 200,000 such cells (from >10 donors for a single host) into Foxp3^+^ progeny upon adoptive transfer in immunodeficient hosts.

While it is possible that TFH cells might simultaneously give rise to both Foxp3^–^ and Foxp3^+^ TR1 subsets, the shared dependency on BLIMP-1, coupled to the observed clonotype sharing and transcriptional/epigenetic similarities between Foxp3+ and Foxp3^–^ TR1 cells relative to their TR1-like and TFH counterparts, makes this scenario highly unlikely. Additionally, our current yet unpublished efforts studying conditional knock-out strains for over 20 different transcription factors have not yielded results that are incompatible with this lineage relationship: in all of the transcription factor knock-out strains where Foxp3^–^ TR1 cells are missing, their Foxp3^+^ TR1 counterparts are missing as well.

As discussed earlier for Foxp3^–^ TR1 cells (7, 13), it is important to note that Foxp3^+^ TR1 cells are not TFR cells (25). Unlike Foxp3^+^ TR1 cells, TFR cells express CXCR5 (but not CCR5) and BCL6 and arise from natural Foxp3^+^ Treg cell precursors. As a result, whereas both *Bcl6* and *Foxp3* deletion can independently impair the development of TFR cells, *Foxp3* is dispensable for pMHCII-NP-induced TR1 cell development (10). Furthermore, whereas deletion of *Prdm1* abrogates pMHCII-NP-induced Foxp3^+^ TR1 cell formation, it enhances the formation of both conventional Foxp3^+^CD25^+^ Treg cells and Foxp3^+^ TFR cells (7).

We had previously shown that selective abrogation of TR1 cell formation or IL-10 expression by TR1 cells were sufficient to abrogate the therapeutic properties of pMOG_38-49_/IA^b^-NPs in B6 mice with EAE (10). That is, pMHCII-NPs lack therapeutic activity in mice in which the pMHCII-NP-induced tetramer^+^ cells lack terminally differentiated TR1 cells (via T cell- or TR1-specific deletion of *Prdm1*) or contain TR1 cells that cannot produce IL-10 (via deletion of *Il10* in TR1 cells) (7). Here we have shown that specific deletion of *Il10* in Foxp3^+^ T cells (via a Foxp3 promoter-driven Cre transgene) had a significant, albeit only partial effect on the therapeutic activity of the anti-encephalitogenic NP compound. Thus, the fullsome therapeutic activity of pMHCII-NPs requires both IL-10-producing Foxp3^–^ and Foxp3^+^ TR1 cells. Work is underway to ascertain the cellular targets of the IL-10 produced by these cell types as well as to determine whether therapeutic activity involves downstream tolerogenic cell types other than IL-10-producing Breg cells (10).

## Methods

### Mice

The strains used herein, along with the cell specificity of the Cre transgenes and the nature of the targeted genes, are listed on **Supplementary Table 2**. NOD/ShiLtJ mice were from the Jackson Lab (Bar Harbor, ME, USA). NOD.*Cd4-Cre* and NOD.*Flpe* mice were produced by backcrossing the *Cd4-Cre* and *Flpe* transgenes from B6.*Cd4-Cre* mice (Tg(Cd4-cre)1Cwi) or B6.*Flpe* mice (B6;SJL-Tg(ACTFLPe)9205Dym/J) onto the NOD.Lt background for at least 10 generations. NOD.*Prdm1^loxP/loxP^* and NOD.*Irf4^loxP/loxP^* mice were produced by backcrossing the *Prdm1^loxP^* and *Irf4^loxP^* genes from *Prdm1^loxP/loxP^* (B6.129-*Prdm*1^tm1Clme^/J), and B6.*Irf4^loxP/loxP^* (B6.129S1-*Irf4^tm1Rdf^*/J) mice, respectively, onto the NOD/ShiLtJ or NOD.*Cd4-Cre* backgrounds for at least five generations, followed by intercrossing.

B6 mice carrying an *Il10^loxP^* allele were generated using the targeted embryonic stem (ES) cell clone EPD0158-4-D-06 from the EuComm consortium (Knockout-First Allele with conditional potential), as described recently (7). B6 mice carrying one copy of an *Il10* ^null^ allele and a copy of the above *Il10* ^loxP^ allele as well as *Tbx21-Cre* o *Foxp3-Cre* transgenes were generated by breeding the various genes from the corresponding B6 stocks (B6.129P2-*Il10^tm1Cgn^*/J, B6.*Il10^loxP/+^*, B6;CBA-Tg(Tbx21-cre)1Dlc/J, and B6.129(Cg)-*Foxp3^tm4(YFP/icre)Ayr^*/J, respectively). B6 mice carrying two copies of a conditional *Prdm1* allele and a *Tbx21-Cre* transgene were generated by introgressing the *Tbx21-Cre* transgene from the B6;CBA-Tg(Tbx21-cre)1Dlc/J stock into B6.129-*Prdm*1^tm1Clme^/J mice.

The experiments described herein were approved by the University of Calgary and Universitat de Barcelona Animal Care Committees.

### pMHC production

Recombinant pMHC class II were produced in CHO-S cells transduced with lentiviruses encoding peptide-MHCα and MHCβ chains and IRES-CFP and IRES-EGFP cassettes, respectively. Transduced CHO cells were grown in 2 L baffled flasks (Nalgene, Thermo Fisher Scientific, Waltham, MA, USA) at 125 rpm, 5% CO_2_ and 37°C. Basal medium was Power-CHO-2 (Lonza, Basel, Switzerland) supplemented with 8 mM Glutamine (Cultek, Madrid, Spain) and Gentamicine Sulfate (0.25 mg/mL) (Lonza). The cultures were supplemented with Cell Boost 7a (Hyclone) at 3% v/v and Cell Boost 7b (Hyclone, GE Healthcare, Chicago, IL, USA) at 0.3% v/v on days 0, 3, 4, 5, 6, 8, 9 and 10. Temperature shift to 34°C was done when cell densities reached 5-7x10^6^ cells/mL. Additional Glutamine was added on day 7, to 2 mM. Glucose was added to 4.5 g/L when levels fell below 3.5 g/L. Cells were harvested on Day 14 or when viability fell below 60%. The secreted proteins were purified by sequential affinity chromatography on nickel and strep-tactin columns and used for NP coating or biotinylated *in vitro* to produce pMHCII tetramers.

### pMHCII tetramers

Phycoerythrin (PE)-conjugated tetramers were prepared using biotinylated pMHCII monomers and used to stain peripheral T-cells. Briefly, pMHCII monomers were subjected to biotinylation using Biotin ligase (Avidity, Aurora, CO, USA) following the supplier’s protocols, followed by ion exchange chromatography using an AKTA FPLC system (GE Healthcare, Chicago, IL, USA). The final product was verified by denaturing SDS-PAGE. Tetramers were generated by adding PE-conjugated streptavidin (Rockland Immunochemicals, Limerick, PA, USA) at a 4:1 molar ratio.

### Flow cytometry

Fluorochrome-conjugated mAbs against mouse CD4 (RM4-5 or GK1.5), B220 (RA3-6B2), and PD-1 (CD279, J43) as well as isotype controls were purchased from BD Biosciences (San Diego, CA, USA). The Abs against ICOS (CD278, C398.4A), CD11b and CD11c were from BioLegend (San Diego, CA). Splenic CD4^+^ T-cells were first incubated with an anti-CD16/CD32 mAb (2.4G2; BD Pharmingen, BD Biosciences, San Diego, CA) for 10 min at room temperature to block FcRs, and then stained with tetramer (5µg/mL) in FACS buffer (0.05% sodium azide and 1% FBS in PBS) for 30 min at 4°C (BDC2.5mi/IA^g7^) or for 90min at 37°C (pMOG_38-49_/IA^b^). Cells were then washed and incubated with FITC- or BV605-conjugated anti-CD4 (5µg/mL), APC-conjugated anti-ICOS, BV421-conjugated anti-PD-1, PerCP-conjugated anti-B220, anti-CD11b and anti-CD11c (2µg/mL; as a ‘dump’ channel) for 30 min at 4°C. Cells were washed again, fixed in 1% paraformaldehyde (PFA) in PBS and analyzed with a BD LSRII flow cytometer. Analysis was done using FlowJo software (FlowJo, BD Biosciences, San Diego, CA, USA).

### Nanoparticle synthesis

Maleimide-functionalized, pegylated iron oxide NPs (PFM series) were produced in a single-step thermal decomposition in the absence of surfactants as described (9). Briefly, 3g Maleimide-PEG (2 kDa MW, Jenkem Tech USA) were melted in a 50mL round bottom flask at 100°C and then mixed with 7 mL of benzyl ether and 2mmol Fe(acac)_3_. The reaction was stirred for 1 hr and heated to 260°C with reflux for 2 hr. The mixture was cooled to room temperature and mixed with 30 mL water. Insoluble materials were removed by centrifugation at 2,000xg for 30 min. The NPs were purified using magnetic (MACS) columns (Miltenyi Biotec, Auburn, CA, USA) and stored in water at room temperature or 4°C. The concentration of iron was determined spectrophotometrically at 410 nm in 2N hydrochloric acid (HCl).

### pMHC conjugation to NPs

pMHC conjugation to maleimide-functionalized NPs (PFM) was done via the free C-terminal Cys engineered into the MHCα chain/knob. Briefly, pMHCs were mixed with NPs in 40 mM phosphate buffer, pH 6.0, containing 2mM ethylenediaminetetraacetic acid (EDTA), 150mM NaCl, and incubated overnight at room temperature. pMHC-conjugated NPs were purified by magnetic separation and concentrated by ultrafiltration through Amicon Ultra-15 (100 kDa cut-off) (Merck KGaA, Darmstadt, Germany) and stored in PBS. **Supplementary Table 2** lists the NP types used in this manuscript, indicating the MHCII allelic type and peptides displayed by each compound.

### NP characterization

The size and dispersity of unconjugated and pMHC-conjugated NPs were assessed via transmission electron microscopy (TEM, Hitachi H7650, Hitachi, Chiyoda, Tokio, Japan) and dynamic light scattering (DLS, Zetasizer, Malvern Panalytical, Spectris, Egham, UK). Pegylated and pMHC-NPs were analyzed via 0.8% agarose gel electrophoresis, native- and denaturing 10% SDS-PAGE. To quantify pMHC valency, we measured the pMHC concentration of the pMHC-NP preps using the Bradford assay (Thermo Scientific).

### pMHCII-NP therapy of NOD mice

Cohorts of 10 week-old female NOD mice were injected i.v. with BDC2.5mi/IA^g7^-coated NPs in PBS twice a week for 5 weeks. Treatment-induced formation and expansion of cognate TR1-like cells were assessed by flow cytometry.

### Pancreatic islet preparation and tetramer staining

Pancreata were injected with ~2 mL collagenase P (Millipore Sigma, 0.66 mg/mL) through the bile duct. They were then digested at 37°C for 15 minutes and dispersed with pipetting. The islets were hand-picked under a stereomicroscope and incubated with IL-2 containing LCM for overnight in a CO_2_ incubator. The islet cells and islet infiltrating mononuclear cells were further treated with trypsin for 3 minutes to make single cell suspensions. For tetramer staining of islet-associated T-cells, after Fc-blocking, cells were stained with BDC2.5mi/IA^g7^ tetramers at 4°C for 30 minutes in the presence of anti-CD4, anti-CD8, anti-CD45R/B220, and a viability dye for the last 15 minutes.

### *In vivo* TR1 cell formation in TFH cell-transfused hosts

We transfused FACS-sorted CXCR5^high^PD-1^high^ CD4^+^ T-cells from the spleens of NOD, NOD.*Cd4-Cre* or NOD.*Cd4-Cre/Prdm1^loxP/loxP^* mice (n=5 mice each) treated with 5 doses of BDC2.5mi/I-A^g7^-NPs (1.5x10^5^/host) into two NOD.*scid* hosts/donor type and treated the hosts with 10 additional doses of pMHCII-NPs. We performed scRNAseq analyses of the sorted CXCR5^high^PD-1^high^ CD4^+^ T-cell pool used for transfer (7), as well as the sorted BDC2.5mi/I-A^g7^ tetramer^+^ CD4^+^ cells arising in the hosts. Cell cluster assignment was done using the scRNAseq data obtained for the pMHCII-NP-induced Tet^+^ cell pools containing the four cell sub-clusters, including the Foxp3^+^ TR1 subset.

### EAE induction and pMHCII-NP therapy

B6 mice (male or female, at least 6 weeks-old) were immunized subcutaneously (under isoflurane anesthesia) on each flank with a total of 150 ug MOG_35-55_ peptide in emulsified in CFA (at a final adjuvant concentration of 1 mg/mL heat-killed *Mycobacterium tuberculosis*, for which the commercial adjuvant was concentrated). Pertussis toxin was administered intravenously on the day of immunization and 3 days after immunization (in PBS, at a dose of 300 ng on each occasion). Daily assessment of the disability scores started on days 7-9 after immunization (depending on when the first animals started to show symptoms), together with daily monitoring of the weight (given as a percentage of the weight on the day of immunization). Scoring for severity of paralysis was performed independently for the tail and each limb, with pMHCII-NP treatment initiated when total score (out of 15) was 4 or higher. Briefly, our scoring system (26, 27) is as follows: Tail: 0 - healthy/fully mobile, 0.5 - fully mobile with slight curve/discoordination, 1 - residual movement (tail only lifted slightly), 1.5 - immobile but with tone, 2 – floppy; Legs (score each): 0- healthy, 0.5 - slightly modified gait without pronounced limping, 1 - limping (no dragging yet), 1.5 - alternates between limping and dragging, 2 - drags feet all the time, still able to exert force and walk, 2.5 - residual movement (i.e. kicking) but not able to walk/bear weight, 3 - paraplegia/full paralysis; Arms (score each): 0 - strong (healthy) grip, 0.5 - slightly weakened grip, 1 - weak grip, 1.5 - weak grip and difficulty extending arm, 2 - unable to grip, can extend (with difficulty), 2.5 - residual movement, 3- full paralysis. Scores are added (tail+ R leg + L leg + R arm + L arm) and the value is divided by 3 to translate from a scale of 15 (raw score) to the reported scores on a 5-point scale. pMHCII-NP treatment groups were randomized into treatment with pMHCII-NP or Cys-capped NP (to ensure initial average scores and weights are as similar as possible) and treatments administered bi-weekly for 10 doses.

### 10x scRNAseq

Cells were partitioned into Gel Bead-In-Emulsions with a Target Cell Recovery of 5.000 total cells. Cell number and viability were verified using a TC20™ Automated Cell Counter (Bio-Rad Laboratories, Hercules, CA, USA). cDNA sequencing libraries were prepared using the NextGEM Single-cell 3’ mRNA kit (V3.1; 10X Genomics) following manufacturer’s instructions. Briefly, after GEM-RT clean up, cDNA was amplified during 13 cycles and cDNA QC and quantification were performed on an Agilent Bioanalyzer High Sensitivity chip (Agilent Technologies). cDNA libraries were indexed by PCR using the PN-220103 Chromiumi7 Sample Index Plate. Size distribution and concentration of 3’ cDNA libraries were verified on an Agilent Bioanalyzer High Sensitivity chip (Agilent Technologies). Finally, sequencing of cDNA libraries was carried out on a NovaSeq 6000 sequencer (Illumina) to obtain approximately 25,000-50,000 paired-end 75bp reads per cell, respectively.

### 10X scRNASeq + full-length V(D)J profiling

Sorted tetramer^+^ cells (10^5^) were processed for 10X scRNASeq 5’ (200 million reads) + full-length V(D)J enrichment (10 million reads) (Chromium Single Cell Immune Profiling), following the supplier’s protocols.

### 10x scMultiome

5x10^5^ live cells were collected in DMEM media (Sigma-Aldrich) supplemented with 10% FBS (Hyclone) at 4°C and processed for single-cell barcoding and library generation following the manufacturer’s instructions (CG000338; 10X Genomics). Briefly, isolated nuclei were partitioned into Gel Bead-In-Emulsions to produce barcoded cDNA from poly-adenylated mRNA as described above, as well as barcoded DNA fragments, and processed for library amplification and sequencing on a NovaSeq 6000 sequencer (Illumina) as described above.

### Bioinformatic analyses

For 10x scRNAseq data, Cell Ranger (version 3.1.0; 10x genomics) was used to process and de-multiplex raw sequencing data. Raw basecall files were first converted to the fastq format, and subsequently the sequences were mapped to the *Mus musculus* genome (version mm10) and demultiplexed to generate single-cell feature counts (using STAR alignment). Downstream analysis including dimensionality reduction (UMAP: Uniform Manifold Approximation and Projection), cluster analysis (K-means), cell type prediction and differential expression analysis, was performed using the package Seurat v4.3 in R. Cell type annotation was performed by projecting the PCA structure of previously annotated pMHCII-NP-induced Tet^+^ T cells from wildtype NOD mice (7) (GSE182636) onto each new query object. Differentially expressed genes were obtained using the function ‘FindMarkers’ using a Wilcoxon Rank Sum test and Bonferroni correction of P values. Integration and batch-effect mitigation of multiple datasets was achieved by canonical correlation analysis (CCA).

For monocle 3 trajectory inference, data were projected into a low-dimensional space using UMAP v.0.3.2. Cells were then clustered using Louvain/Leiden community detection to generate a principal graph using an embedding procedure based on the SimplePPT algorithm, which was used as a guide for pseudotime computing.

Single-cell RNA+ATAC multiome demultiplexing, alignment and filtering of raw sequencing data as well as subsequent barcode counting (UMIs), and peak calling was done using Cell Ranger ARC (v2.0) software pipelines (10X genomics). For alignment steps, these pipelines use STAR (28) and BWA (29). The mouse genome assembly GRCm38(mm10) was used as the reference. Downstream analysis of gene/fragment-cell matrices was done using Seurat v4.3 (30) and Signac v1.3.0 packages (31). Briefly, low-quality cells were filtered out based on mitochondrial DNA content and UMIs count. Both RNA and ATAC was normalized, scaled, and dimensionally reduced. Multimodal analysis was performed using a WNN (Weighted Nearest Neighbor) analysis. UMAP was used for visualization and cell clustering. Differentially expressed genes or enriched chromatin sites were determined using the function ‘FindMarkers’ using a Wilcoxon Rank Sum test for RNA data and Likelihood ratio test for ATAC data, applying a log2 fold change threshold of 0.25 and Bonferroni correction of P values.

Cell subsets were identified via prediction annotation using wildtype control dataset as a reference. This reference annotation was applied consistently across the study to ensure objectivity and reproducibility in cell type identification and analysis.

### Enrichment pathway analyses

Differentially expressed genes (|FC|>2 and FDR<0.05) were obtained from 10X genomics-based scRNAseq data corresponding to Foxp3^+^ vs Foxp3- TR1 sub-clusters within sorted Tet^+^ cells from NOD mice treated with BDC2.5mi/IA^g7^-NPs. Enrichment analysis was performed using Gene Set enrichment analysis of Gene Ontology (gseGO) of `clusterProfiler` R package (adjusted P-value < 0.05).

### Statistical analyses

Unless specified, sample size values mentioned in the figure legends correspond to the total number of samples examined. Data were compared in GraphPad Prism versions 6-9 using Mann-Whitney *U*-test or two-way ANOVA. P values <0.05 were considered statistically significant. Only statistically significant P values are displayed on Figures.

## Data and code availability

All unique stable cell lines/reagents generated in this study are available from the corresponding authors with a completed Material Transfer Agreement. The raw RNAseq and scRNAseq data files have been uploaded into the GEO database under the following accession numbers: GSE182636 (scRNAseq data, week 0 pMHCII-NP-induced Tet^+^ T cells in NOD and NOD.CD4-Cre.Prdm1loxP/loxP mice); GSE248152 (scMultiome data); GSE252235 (scRNAseq data of BDC2.5mi/IAg7-NP-induced Tet^+^ T cells in NOD mice post-treatment withdrawal, TFH-transfused NOD.Scid hosts, NOD pancreatic islet-associated T cells and BDC2.5mi/IAg7-NP-induced Tet^+^ T cells from NOD.CD4-Cre.Irf4loxP/loxP mice, and scRNAseq 10x VDJ immunoprofiling datasets).

## Data Availability

The datasets presented in this study can be found in online repositories. The names of the repository/repositories and accession number(s) can be found in the article/**Supplementary Material**.
