# Supplementary material for: BLIMP-1-dependent differentiation of T follicular helper cells into Foxp3+ T regulatory type 1 cells
Source: Front Immunol. 2025 Feb 24;16:1519780. doi: 10.3389/fimmu.2025.1519780 (PMC11891242; doi:10.3389/fimmu.2025.1519780)
Supplement: Supplementary file 12 [file SupplementaryFile1.pdf]

Supplementary Table 1. Expression of 106 TR1/TFH/Treg-relevant genes in TFH, TR1 and conventional Foxp3+ Treg cell types

Gene type	Genes (protein)	Described in TR1 (ref)	Described in other Treg types (ref)	Described in TFH (ref)
Cell adhesion molecules	Cd226	+ (1)	ND	ND
	Itga2 (CD49b)	+ (1)	ND	ND
	Itgae (CD103)	+ (2)	+ (3)	ND
	Ocln	ND	ND	ND
	S1pr2	ND	ND	+ (4)
	Sell (CD62L)	- (5)	+ (6)	- (7)
	Selplg (Psgl1)	ND	ND	- (8)
Chemokyne receptors	Ccr5	+ (9)	+ (10)	+ (11)
	Ccr7	ND	+ (12)	- (13)
	Cxcr3	+ (14)	+ (12)	In some subsets (15)
	Cxcr4	ND	ND	+ (16)
	Cxcr5	ND	ND	+ (17)
Co-estimulatory molecules	Cd28	+ (18)	ND	+ (19)
	Cd40lg	ND	ND	+ (20)
	Icos	+ (21)	+ (22)	+ (23)
	Klrk1 (NKG2D)	ND	+ (24)	ND
	Sh2d1a (SAP)	ND	ND	+ (25)
	Tnfrsf4 (Ox40)	+ (26)	+ (27)	+ (28)
	Tnfrsf18 (GITR)	+ (18)	+ (29)	ND
	Tnfsf4 (Ox40L)	ND	ND	ND
Co-inhibitory molecules	Ctla4	+ (30)	+ (31)	+ (32)
	Fasl	ND	+ (33)	ND
	Havcr2 (TIM-3)	+ (34)	+ (35)	+ (36)
	Lag3	+ (1)	+ (37)	ND
	Pdcd1 (PD-1)	+ (9)	+ (38)	+ (17)
	Tigit	+ (39)	+ (40)	+ (41)
Cytokines	Ebi3 (IL27b)	ND	+ (42)	ND
	Ifng	+ (43)	ND	ND
	Il10	+ (44)	+ (45)	+ (46)
	Il21	+ (47)	ND	+ (48)
	Il4	- (44)	ND	+ (49)
	Il5	+ (44)	ND	ND
	Mcub (Areg)	+	+ (50)	ND
	Tgfb1	+ (44)	+ (45)	ND
Cytokine receptors	Il10ra	+ (51)	+ (52)	ND
	Il12rb2	ND	+ (53)	ND
	Il21r	+ (47)	+ (54)	+ (48)
	Il27ra	+ (55)	+ (56)	+ (57)
	IL7r (CD127)	- (21)	- (58)	- (59)
	IL17rc	ND	ND	ND
	Il2ra (CD25)	- (18)	+ (60)	- (61)
	Tgfbr1	- (51)	+ (62)	ND
	Tgfbr2	- (51)	+ (63)	ND
	Tgfbr3	- (51)	ND	ND
	Atf6	- (51)	ND	ND
	Ahr	+ (64)	ND	ND
	Ajuba	ND	ND	ND
	Ascl2	ND	ND	+ (65)
	Bach2	- (66)	+ (67)	- (68)
	Batf	+ (69)	ND	+ (70)
	Bcl6	ND	ND	+ (71)
	Bhlhe40	+ (51)	ND	ND
	Bmyc	- (51)	ND	ND

Transcription factors	Cbfa2t3	ND	ND	ND
	Cebpa	ND	ND	+ (72)
	Dbp	- (51)	ND	ND
	E2f1	+ (51)	+ (73)	ND
	Egr2	+ (74)	ND	ND
	Elk4	- (51)	ND	ND
	Eomes	+ (75)	+ (76)	ND
	FoxP1	ND	ND	- (77)
	FoxP3	ND	+ (78)	ND
	Grhl1	ND	ND	ND
	Hmgb2	+ (51)	ND	ND
	Id2	+ (51)	+ (79)	- (80)
	Id3	- (51)	+ (79)	+ (80)
	Irf1	+ (69)	ND	ND
	Irf4	+ (81)	+ (3)	+ (82)
	Jdp2	ND	ND	ND
	Klf2	ND	ND	- (83)
	Lef1	ND	ND	+ (84)
	Lilrb4a	ND	+ (85)	ND
	Maf	+ (47)	ND	+ (86)
	Myb	- (51)	+ (87)	ND
	Mybl2	+ (51)	ND	ND
	Myc	- (51)	- (88)	ND
	Nfia	ND	ND	ND
	Nfil3	+ (34)	ND	ND
	Nr1h3 (LXRα)	+ (51)	+ (89)	ND
	Pax5	ND	ND	ND
	Pax9	ND	ND	ND
	Pou2af1 (OcaB)	ND	ND	ND
	Prdm1 (Blimp-1)	+ (90)	ND	+ (91)
	Rbpj	+ (51)	+ (92)	ND
	Runx2	+ (51)	ND	ND
	Rora	+ (51)	ND	- (93)
	S1pr1	ND	ND	- (83)
	Six5	ND	ND	ND
	Sox4	- (51)	- (94)	ND
	Sox8	ND	ND	ND
	Stat1	+ (95)	ND	+ (96)
	Stat3	+ (97)	ND	+ (23)
	Stat4	ND	- (98)	+ (99)
	Tbx21 (T-bet)	+ (75)	ND	- (84)
	Tcf7	ND	ND	+ (84)
	Tox2	ND	ND	+ (80)
	Vdr	ND	+ (100)	ND
	Zbtb16 (PLZF)	+ (51)	ND	ND
Secretion proteins	Chgb	ND	ND	+ (101)
	Gzmb (Granzyme B)	+ (102)	+ (103)	ND
Enzymes	Cblb	ND	+ (104)	ND
	Entpd1 (CD39)	+ (105)	+ (106)	ND
	Itk	+ (107)	+ (108)	ND
	Nt5e (CD73)	+ (105)	+ (106)	+ (109)
	Serpinb6b	+ (51)	+ (110)	ND
	Serpinb9	+ (51)	ND	ND

References for Suppl. Table 1

1. N. Gagliani, C. F. Magnani, S. Huber, M. E. Gianolini, M. Pala, P. Licona-Limon, B. Guo, D. R. Herbert, A. Bulfone, F. Trentini, C. Di Serio, R. Bacchetta, M. Andreani, L. Brockmann, S. Gregori, R. A. Flavell, M. G. Roncarolo, Coexpression of CD49b and LAG-3 identifies human and mouse T regulatory type 1 cells. *Nat. Med.* **19**, 739-746 (2013).
2. W. Duan, T. So, A. K. Mehta, H. Choi, M. Croft, Inducible CD4+LAP+Foxp3- regulatory T cells suppress allergic inflammation. *J. Immunol.* **187**, 6499-6507 (2011).
3. E. Cretney, A. Xin, W. Shi, M. Minnich, F. Masson, M. Miasari, G. T. Belz, G. K. Smyth, M. Busslinger, S. L. Nutt, A. Kallies, The transcription factors Blimp-1 and IRF4 jointly control the differentiation and function of effector regulatory T cells. *Nat. Immunol.* **12**, 304-311 (2011).
4. S. Moriyama, N. Takahashi, J. A. Green, S. Hori, M. Kubo, J. G. Cyster, T. Okada, Sphingosine-1-phosphate receptor 2 is critical for follicular helper T cell retention in germinal centers. *J. Exp. Med.* **211**, 1297-1305 (2014).
5. P. L. Bollyky, R. P. Wu, B. A. Falk, J. D. Lord, S. A. Long, A. Preisinger, B. Teng, G. E. Holt, N. E. Standifer, K. R. Braun, C. F. Xie, P. L. Samuels, R. B. Vernon, J. A. Gebe, T. N. Wight, G. T. Nepom, ECM components guide IL-10 producing regulatory T-cell (TR1) induction from effector memory T-cell precursors. *Proc. Natl. Acad. Sci. U. S. A.* **108**, 7938-7943 (2011).
6. M. Biswas, S. R. P. Kumar, C. Terhorst, R. W. Herzog, Gene Therapy With Regulatory T Cells: A Beneficial Alliance. *Front. Immunol.* **9**, 554 (2018).
7. N. Fazilleau, L. J. McHeyzer-Williams, H. Rosen, M. G. McHeyzer-Williams, The function of follicular helper T cells is regulated by the strength of T cell antigen receptor binding. *Nat. Immunol.* **10**, 375-384 (2009).
8. A. C. Poholek, K. Hansen, S. G. Hernandez, D. Eto, A. Chandele, J. S. Weinstein, X. Dong, J. M. Odegard, S. M. Kaech, A. L. Dent, S. Crotty, J. Craft, In vivo regulation of Bcl6 and T follicular helper cell development. *J. Immunol.* **185**, 313-326 (2010).
9. J. S. Alfen, P. Larghi, F. Facciotti, N. Gagliani, R. Bosotti, M. Paroni, S. Maglie, P. Gruarin, C. M. Vasco, V. Ranzani, C. Frusteri, A. Iseppon, M. Moro, M. C. Crosti, S. Gatti, M. Pagani, F. Caprioli, S. Abrignani, R. A. Flavell, J. Geginat, Intestinal IFN-gamma-producing type 1 regulatory T cells coexpress CCR5 and programmed cell death protein 1 and downregulate IL-10 in the inflamed guts of patients with inflammatory bowel disease. *J. Allergy Clin. Immunol.* **142**, 1537-1547 e1538 (2018).
10. R. S. Bystry, V. Aluvihare, K. A. Welch, M. Kallikourdis, A. G. Betz, B cells and professional APCs recruit regulatory T cells via CCL4. *Nat. Immunol.* **2**, 1126-1132 (2001).
11. S. M. Miller, B. Miles, K. Guo, J. Folkvord, A. L. Meditz, M. D. McCarter, D. N. Levy, S. MaWhinney, M. L. Santiago, E. Connick, Follicular Regulatory T Cells Are Highly Permissive to R5-Tropic HIV-1. *J. Virol.* **91**, (2017).
12. K. S. Smigiel, E. Richards, S. Srivastava, K. R. Thomas, J. C. Dudda, K. D. Klonowski, D. J. Campbell, CCR7 provides localized access to IL-2 and defines homeostatically distinct regulatory T cell subsets. *J. Exp. Med.* **211**, 121-136 (2014).
13. N. M. Haynes, C. D. Allen, R. Lesley, K. M. Ansel, N. Killeen, J. G. Cyster, Role of CXCR5 and CCR7 in follicular Th cell positioning and appearance of a programmed cell death gene-1high germinal center-associated subpopulation. *J. Immunol.* **179**, 5099-5108 (2007).

14. M. A. Kunicki, L. C. Amaya Hernandez, K. L. Davis, R. Bacchetta, M. G. Roncarolo, Identity and Diversity of Human Peripheral Th and T Regulatory Cells Defined by Single-Cell Mass Cytometry. *J. Immunol.* **200**, 336-346 (2018).
15. N. Chevalier, Quantifying helper cell function of human TFH cells in vitro. *Methods Mol. Biol.* **1291**, 209-226 (2015).
16. C. D. Allen, K. M. Ansel, C. Low, R. Lesley, H. Tamamura, N. Fujii, J. G. Cyster, Germinal center dark and light zone organization is mediated by CXCR4 and CXCR5. *Nat. Immunol.* **5**, 943-952 (2004).
17. S. Crotty, Follicular helper CD4 T cells (TFH). *Annu. Rev. Immunol.* **29**, 621-663 (2011).
18. H. Zeng, R. Zhang, B. Jin, L. Chen, Type 1 regulatory T cells: a new mechanism of peripheral immune tolerance. *Cell. Mol. Immunol.* **12**, 566-571 (2015).
19. M. A. Linterman, A. E. Denton, D. P. Divekar, I. Zvetkova, L. Kane, C. Ferreira, M. Veldhoen, S. Clare, G. Dougan, M. Espeli, K. G. Smith, CD28 expression is required after T cell priming for helper T cell responses and protective immunity to infection. *Elife* **3**, (2014).
20. C. G. Vinuesa, S. G. Tangye, B. Moser, C. R. Mackay, Follicular B helper T cells in antibody responses and autoimmunity. *Nat. Rev. Immunol.* **5**, 853-865 (2005).
21. B. Haringer, L. Lozza, B. Steckel, J. Geginat, Identification and characterization of IL-10/IFN-gamma-producing effector-like T cells with regulatory function in human blood. *J. Exp. Med.* **206**, 1009-1017 (2009).
22. L. Strauss, C. Bergmann, M. J. Szczepanski, S. Lang, J. M. Kirkwood, T. L. Whiteside, Expression of ICOS on human melanoma-infiltrating CD4+CD25highFoxp3+ T regulatory cells: implications and impact on tumor-mediated immune suppression. *J. Immunol.* **180**, 2967-2980 (2008).
23. R. I. Nurieva, Y. Chung, D. Hwang, X. O. Yang, H. S. Kang, L. Ma, Y. H. Wang, S. S. Watowich, A. M. Jetten, Q. Tian, C. Dong, Generation of T follicular helper cells is mediated by interleukin-21 but independent of T helper 1, 2, or 17 cell lineages. *Immunity* **29**, 138-149 (2008).
24. K. Fujio, T. Okamura, K. Yamamoto, The Family of IL-10-secreting CD4+ T cells. *Adv. Immunol.* **105**, 99-130 (2010).
25. S. Crotty, E. N. Kersh, J. Cannons, P. L. Schwartzberg, R. Ahmed, SAP is required for generating long-term humoral immunity. *Nature* **421**, 282-287 (2003).
26. T. Ito, Y. H. Wang, O. Duramad, S. Hanabuchi, O. A. Perng, M. Gilliet, F. X. Qin, Y. J. Liu, OX40 ligand shuts down IL-10-producing regulatory T cells. *Proc. Natl. Acad. Sci. U. S. A.* **103**, 13138-13143 (2006).
27. J. D. Fontenot, J. P. Rasmussen, L. M. Williams, J. L. Dooley, A. G. Farr, A. Y. Rudensky, Regulatory T cell lineage specification by the forkhead transcription factor foxp3. *Immunity* **22**, 329-341 (2005).
28. C. Jacquemin, N. Schmitt, C. Contin-Bordes, Y. Liu, P. Narayanan, J. Seneschal, T. Maurouard, D. Dougall, E. S. Davizon, H. Dumortier, I. Douchet, L. Raffray, C. Richez, E. Lazaro, P. Duffau, M. E. Truchetet, L. Khoryati, P. Mercie, L. Couzi, P. Merville, T. Schaeverbeke, J. F. Viallard, J. L. Pellegrin, J. F. Moreau, S. Muller, S. Zurawski, R. L. Coffman, V. Pascual, H. Ueno, P. Blanco, OX40 Ligand Contributes to Human Lupus Pathogenesis by Promoting T Follicular Helper Response. *Immunity* **42**, 1159-1170 (2015).
29. J. Shimizu, S. Yamazaki, T. Takahashi, Y. Ishida, S. Sakaguchi, Stimulation of CD25(+)CD4(+) regulatory T cells through GITR breaks immunological self-tolerance. *Nat. Immunol.* **3**, 135-142 (2002).

30. R. Bacchetta, C. Sartirana, M. K. Levings, C. Bordignon, S. Narula, M. G. Roncarolo, Growth and expansion of human T regulatory type 1 cells are independent from TCR activation but require exogenous cytokines. *Eur. J. Immunol.* **32**, 2237-2245 (2002).
31. L. S. Walker, Treg and CTLA-4: two intertwining pathways to immune tolerance. *J. Autoimmun.* **45**, 49-57 (2013).
32. P. T. Sage, A. M. Paterson, S. B. Lovitch, A. H. Sharpe, The coinhibitory receptor CTLA-4 controls B cell responses by modulating T follicular helper, T follicular regulatory, and T regulatory cells. *Immunity* **41**, 1026-1039 (2014).
33. A. V. Gorbachev, R. L. Fairchild, CD4+CD25+ regulatory T cells utilize FasL as a mechanism to restrict DC priming functions in cutaneous immune responses. *Eur. J. Immunol.* **40**, 2006-2015 (2010).
34. C. Zhu, K. Sakuishi, S. Xiao, Z. Sun, S. Zaghouani, G. Gu, C. Wang, D. J. Tan, C. Wu, M. Rangachari, T. Pertel, H. T. Jin, R. Ahmed, A. C. Anderson, V. K. Kuchroo, An IL-27/NFIL3 signalling axis drives Tim-3 and IL-10 expression and T-cell dysfunction. *Nature communications* **6**, 6072 (2015).
35. S. Gupta, T. B. Thornley, W. Gao, R. Larocca, L. A. Turka, V. K. Kuchroo, T. B. Strom, Allograft rejection is restrained by short-lived TIM-3+PD-1+Foxp3+ Tregs. *J. Clin. Invest.* **122**, 2395-2404 (2012).
36. S. Zhu, J. Lin, G. Qiao, X. Wang, Y. Xu, Tim-3 identifies exhausted follicular helper T cells in breast cancer patients. *Immunobiology* **221**, 986-993 (2016).
37. C. T. Huang, C. J. Workman, D. Flies, X. Pan, A. L. Marson, G. Zhou, E. L. Hipkiss, S. Ravi, J. Kowalski, H. I. Levitsky, J. D. Powell, D. M. Pardoll, C. G. Drake, D. A. Vignali, Role of LAG-3 in regulatory T cells. *Immunity* **21**, 503-513 (2004).
38. X. Chen, D. Fosco, D. E. Kline, L. Meng, S. Nishi, P. A. Savage, J. Kline, PD-1 regulates extrathymic regulatory T-cell differentiation. *Eur. J. Immunol.* **44**, 2603-2616 (2014).
39. B. R. Burton, G. J. Britton, H. Fang, J. Verhagen, B. Smithers, C. A. Sabatos-Peyton, L. J. Carney, J. Gough, S. Strobel, D. C. Wraith, Sequential transcriptional changes dictate safe and effective antigen-specific immunotherapy. *Nature communications* **5**, 4741 (2014).
40. X. Yu, K. Harden, L. C. Gonzalez, M. Francesco, E. Chiang, B. Irving, I. Tom, S. Ivelja, C. J. Refino, H. Clark, D. Eaton, J. L. Grogan, The surface protein TIGIT suppresses T cell activation by promoting the generation of mature immunoregulatory dendritic cells. *Nat. Immunol.* **10**, 48-57 (2009).
41. E. Godefroy, H. Zhong, P. Pham, D. Friedman, K. Yazdanbakhsh, TIGIT-positive circulating follicular helper T cells display robust B-cell help functions: potential role in sickle cell alloimmunization. *Haematologica* **100**, 1415-1425 (2015).
42. L. W. Collison, C. J. Workman, T. T. Kuo, K. Boyd, Y. Wang, K. M. Vignali, R. Cross, D. Sehy, R. S. Blumberg, D. A. A. Vignali, The inhibitory cytokine IL-35 contributes to regulatory T-cell function. *Nature* **450**, 566-569 (2007).
43. F. Facciotti, N. Gagliani, B. Haringer, J. S. Alfen, A. Penatti, S. Maglie, M. Paroni, A. Iseppon, M. Moro, M. C. Crosti, K. Stolzel, C. Romagnani, G. Moroni, F. Ingegnoli, S. Torretta, L. Pignataro, A. Annoni, F. Russo, M. Pagani, S. Abrignani, P. Meroni, R. Flavell, J. Geginat, IL-10-producing forkhead box protein 3-negative regulatory T cells inhibit B-cell responses and are involved in systemic lupus erythematosus. *J. Allergy Clin. Immunol.* **137**, 318-321 e315 (2016).

44. H. Groux, A. O'Garra, M. Bigler, M. Rouleau, S. Antonenko, J. E. de Vries, M. G. Roncarolo, A CD4+ T-cell subset inhibits antigen-specific T-cell responses and prevents colitis. *Nature* **389**, 737-742 (1997).
45. H. Liu, B. Hu, D. Xu, F. Y. Liew, CD4+CD25+ regulatory T cells cure murine colitis: the role of IL-10, TGF-beta, and CTLA4. *J. Immunol.* **171**, 5012-5017 (2003).
46. Y. Zhu, L. Zou, Y. C. Liu, T follicular helper cells, T follicular regulatory cells and autoimmunity. *Int. Immunol.* **28**, 173-179 (2016).
47. C. Pot, H. Jin, A. Awasthi, S. M. Liu, C. Y. Lai, R. Madan, A. H. Sharpe, C. L. Karp, S. C. Miaw, I. C. Ho, V. K. Kuchroo, Cutting edge: IL-27 induces the transcription factor c-Maf, cytokine IL-21, and the costimulatory receptor ICOS that coordinately act together to promote differentiation of IL-10-producing Tr1 cells. *J. Immunol.* **183**, 797-801 (2009).
48. T. Chtanova, S. G. Tangye, R. Newton, N. Frank, M. R. Hodge, M. S. Rolph, C. R. Mackay, T follicular helper cells express a distinctive transcriptional profile, reflecting their role as non-Th1/Th2 effector cells that provide help for B cells. *J. Immunol.* **173**, 68-78 (2004).
49. I. L. King, M. Mohrs, IL-4-producing CD4+ T cells in reactive lymph nodes during helminth infection are T follicular helper cells. *J. Exp. Med.* **206**, 1001-1007 (2009).
50. D. Burzyn, W. Kuswanto, D. Kolodin, J. L. Shadrach, M. Cerletti, Y. Jang, E. Sefik, T. G. Tan, A. J. Wagers, C. Benoist, D. Mathis, A special population of regulatory T cells potentiates muscle repair. *Cell* **155**, 1282-1295 (2013).
51. L. Brockmann, S. Soukou, B. Steglich, P. Czarnewski, L. Zhao, S. Wende, T. Bedke, C. Ergen, C. Manthey, T. Agalioti, M. Geffken, O. Seiz, S. M. Parigi, C. Sorini, J. Geginat, K. Fujio, T. Jacobs, T. Roesch, J. R. Izbicki, A. W. Lohse, R. A. Flavell, C. Krebs, J. A. Gustafsson, P. Antonson, M. G. Roncarolo, E. J. Villablanca, N. Gagliani, S. Huber, Molecular and functional heterogeneity of IL-10-producing CD4(+) T cells. *Nature communications* **9**, 5457 (2018).
52. P. Diefenhardt, A. Nosko, M. A. Kluger, J. V. Richter, C. Wegscheid, Y. Kobayashi, G. Tiegs, S. Huber, R. A. Flavell, R. A. K. Stahl, O. M. Steinmetz, IL-10 Receptor Signaling Empowers Regulatory T Cells to Control Th17 Responses and Protect from GN. *J. Am. Soc. Nephrol.* **29**, 1825-1837 (2018).
53. Z. Zhao, S. Yu, D. C. Fitzgerald, M. Elbehi, B. Ciric, A. M. Rostami, G. X. Zhang, IL-12R beta 2 promotes the development of CD4+CD25+ regulatory T cells. *J. Immunol.* **181**, 3870-3876 (2008).
54. A. Comes, O. Rosso, A. M. Orengo, E. Di Carlo, C. Sorrentino, R. Meazza, T. Piazza, B. Valzasina, P. Nanni, M. P. Colombo, S. Ferrini, CD25+ regulatory T cell depletion augments immunotherapy of micrometastases by an IL-21-secreting cellular vaccine. *J. Immunol.* **176**, 1750-1758 (2006).
55. R. R. Meka, S. H. Venkatesha, S. Dudics, B. Acharya, K. D. Moudgil, IL-27-induced modulation of autoimmunity and its therapeutic potential. *Autoimmun Rev* **14**, 1131-1141 (2015).
56. A. V. Villarino, J. Larkin, 3rd, C. J. Saris, A. J. Caton, S. Lucas, T. Wong, F. J. de Sauvage, C. A. Hunter, Positive and negative regulation of the IL-27 receptor during lymphoid cell activation. *J. Immunol.* **174**, 7684-7691 (2005).
57. M. Batten, N. Ramamoorthi, N. M. Kljavin, C. S. Ma, J. H. Cox, H. S. Dengler, D. M. Danilenko, P. Caplazi, M. Wong, D. A. Fulcher, M. C. Cook, C. King, S. G. Tangye, F. J. de Sauvage, N. Ghilardi, IL-27 supports germinal center function by enhancing IL-21 production and the function of T follicular helper cells. *J. Exp. Med.* **207**, 2895-2906 (2010).

58. W. Liu, A. L. Putnam, Z. Xu-Yu, G. L. Szot, M. R. Lee, S. Zhu, P. A. Gottlieb, P. Kapranov, T. R. Gingeras, B. Fazekas de St Groth, C. Clayberger, D. M. Soper, S. F. Ziegler, J. A. Bluestone, CD127 expression inversely correlates with FoxP3 and suppressive function of human CD4+ T reg cells. *J. Exp. Med.* **203**, 1701-1711 (2006).
59. P. W. McDonald, K. A. Read, C. E. Baker, A. E. Anderson, M. D. Powell, A. Ballesteros-Tato, K. J. Oestreich, IL-7 signalling represses Bcl-6 and the TFH gene program. *Nature communications* **7**, 10285 (2016).
60. S. Sakaguchi, N. Sakaguchi, M. Asano, M. Itoh, M. Toda, Immunologic self-tolerance maintained by activated T cells expressing IL-2 receptor alpha-chains (CD25). Breakdown of a single mechanism of self-tolerance causes various autoimmune diseases. *J. Immunol.* **155**, 1151-1164 (1995).
61. A. Ballesteros-Tato, B. Leon, B. A. Graf, A. Moquin, P. S. Adams, F. E. Lund, T. D. Randall, Interleukin-2 inhibits germinal center formation by limiting T follicular helper cell differentiation. *Immunity* **36**, 847-856 (2012).
62. Y. Liu, P. Zhang, J. Li, A. B. Kulkarni, S. Perruche, W. Chen, A critical function for TGF-beta signaling in the development of natural CD4+CD25+Foxp3+ regulatory T cells. *Nat. Immunol.* **9**, 632-640 (2008).
63. W. Ouyang, O. Beckett, Q. Ma, M. O. Li, Transforming growth factor-beta signaling curbs thymic negative selection promoting regulatory T cell development. *Immunity* **32**, 642-653 (2010).
64. L. Apetoh, F. J. Quintana, C. Pot, N. Joller, S. Xiao, D. Kumar, E. J. Burns, D. H. Sherr, H. L. Weiner, V. K. Kuchroo, The aryl hydrocarbon receptor interacts with c-Maf to promote the differentiation of type 1 regulatory T cells induced by IL-27. *Nat. Immunol.* **11**, 854-861 (2010).
65. X. Liu, X. Chen, B. Zhong, A. Wang, X. Wang, F. Chu, R. I. Nurieva, X. Yan, P. Chen, L. G. van der Flier, H. Nakatsukasa, S. S. Neelapu, W. Chen, H. Clevers, Q. Tian, H. Qi, L. Wei, C. Dong, Transcription factor achaete-scute homologue 2 initiates follicular T-helper-cell development. *Nature* **507**, 513-518 (2014).
66. C. L. Edwards, M. M. de Oca, F. de Labastida Rivera, R. Kumar, S. S. Ng, Y. Wang, F. H. Amante, K. Kometani, T. Kurosaki, T. Sidwell, A. Kallies, C. R. Engwerda, The Role of BACH2 in T Cells in Experimental Malaria Caused by Plasmodium chabaudi chabaudi AS. *Front. Immunol.* **9**, 2578 (2018).
67. F. M. Grant, J. Yang, R. Nasrallah, J. Clarke, F. Sadiyah, S. K. Whiteside, C. J. Imianowski, P. Kuo, P. Vardaka, T. Todorov, N. Zandhuis, I. Patrascan, D. F. Tough, K. Kometani, R. Eil, T. Kurosaki, K. Okkenhaug, R. Roychoudhuri, BACH2 drives quiescence and maintenance of resting Treg cells to promote homeostasis and cancer immunosuppression. *J. Exp. Med.* **217**, (2020).
68. A. Lahmann, J. Kuhrau, F. Fuhrmann, F. Heinrich, L. Bauer, P. Durek, M. F. Mashreghi, A. Hutloff, Bach2 Controls T Follicular Helper Cells by Direct Repression of Bcl-6. *J. Immunol.* **202**, 2229-2239 (2019).
69. K. Karwacz, E. R. Miraldi, M. Pokrovskii, A. Madi, N. Yosef, I. Wortman, X. Chen, A. Watters, N. Carriero, A. Awasthi, A. Regev, R. Bonneau, D. Littman, V. K. Kuchroo, Critical role of IRF1 and BATF in forming chromatin landscape during type 1 regulatory cell differentiation. *Nat. Immunol.* **18**, 412-421 (2017).
70. W. Ise, M. Kohyama, B. U. Schraml, T. Zhang, B. Schwer, U. Basu, F. W. Alt, J. Tang, E. M. Oltz, T. L. Murphy, K. M. Murphy, The transcription factor BATF controls the global regulators of class-switch recombination in both B cells and T cells. *Nat. Immunol.* **12**, 536-543 (2011).

71. R. I. Nurieva, Y. Chung, G. J. Martinez, X. O. Yang, S. Tanaka, T. D. Matskevitch, Y. H. Wang, C. Dong, Bcl6 mediates the development of T follicular helper cells. *Science* **325**, 1001-1005 (2009).
72. S. Tanaka, K. Tanaka, F. Magnusson, Y. Chung, G. J. Martinez, Y. H. Wang, R. I. Nurieva, T. Kurosaki, C. Dong, CCAAT/enhancer-binding protein alpha negatively regulates IFN-gamma expression in T cells. *J. Immunol.* **193**, 6152-6160 (2014).
73. R. Bao, X. Shui, J. Hou, J. Li, X. Deng, X. Zhu, T. Yang, Adenosine and the adenosine A2A receptor agonist, CGS21680, upregulate CD39 and CD73 expression through E2F-1 and CREB in regulatory T cells isolated from septic mice. *Int. J. Mol. Med.* **38**, 969-975 (2016).
74. T. Okamura, K. Fujio, M. Shibuya, S. Sumitomo, H. Shoda, S. Sakaguchi, K. Yamamoto, CD4+CD25-LAG3+ regulatory T cells controlled by the transcription factor Egr-2. *Proc. Natl. Acad. Sci. U. S. A.* **106**, 13974-13979 (2009).
75. P. Zhang, J. S. Lee, K. H. Gartlan, I. S. Schuster, I. Comerford, A. Varelias, M. A. Ullah, S. Vuckovic, M. Koyama, R. D. Kuns, K. R. Locke, K. J. Beckett, S. D. Olver, L. D. Samson, M. Montes de Oca, F. de Labastida Rivera, A. D. Clouston, G. T. Belz, B. R. Blazar, K. P. MacDonald, S. R. McColl, R. Thomas, C. R. Engwerda, M. A. Degli-Esposti, A. Kallies, S. K. Tey, G. R. Hill, Eomesodermin promotes the development of type 1 regulatory T (TR1) cells. *Sci Immunol* **2**, (2017).
76. D. A. Schaer, S. Budhu, C. Liu, C. Bryson, N. Malandro, A. Cohen, H. Zhong, X. Yang, A. N. Houghton, T. Merghoub, J. D. Wolchok, GITR pathway activation abrogates tumor immune suppression through loss of regulatory T cell lineage stability. *Cancer Immunol Res* **1**, 320-331 (2013).
77. H. Wang, J. Geng, X. Wen, E. Bi, A. V. Kossenkov, A. I. Wolf, J. Tas, Y. S. Choi, H. Takata, T. J. Day, L. Y. Chang, S. L. Sprout, E. K. Becker, J. Willen, L. Tian, X. Wang, C. Xiao, P. Jiang, S. Crotty, G. D. Victora, L. C. Showe, H. O. Tucker, J. Erikson, H. Hu, The transcription factor Foxp1 is a critical negative regulator of the differentiation of follicular helper T cells. *Nat. Immunol.* **15**, 667-675 (2014).
78. S. Hori, T. Nomura, S. Sakaguchi, Control of regulatory T cell development by the transcription factor Foxp3. *Science* **299**, 1057-1061 (2003).
79. M. Miyazaki, K. Miyazaki, S. Chen, M. Itoi, M. Miller, L. F. Lu, N. Varki, A. N. Chang, D. H. Broide, C. Murre, Id2 and Id3 maintain the regulatory T cell pool to suppress inflammatory disease. *Nat. Immunol.* **15**, 767-776 (2014).
80. Y. S. Choi, J. A. Yang, I. Yusuf, R. J. Johnston, J. Greenbaum, B. Peters, S. Crotty, Bcl6 expressing follicular helper CD4 T cells are fate committed early and have the capacity to form memory. *J. Immunol.* **190**, 4014-4026 (2013).
81. J. O. Jin, X. Han, Q. Yu, Interleukin-6 induces the generation of IL-10-producing Tr1 cells and suppresses autoimmune tissue inflammation. *J. Autoimmun.* **40**, 28-44 (2013).
82. H. Kwon, D. Thierry-Mieg, J. Thierry-Mieg, H. P. Kim, J. Oh, C. Tunyaplin, S. Carotta, C. E. Donovan, M. L. Goldman, P. Tailor, K. Ozato, D. E. Levy, S. L. Nutt, K. Calame, W. J. Leonard, Analysis of interleukin-21-induced Prdm1 gene regulation reveals functional cooperation of STAT3 and IRF4 transcription factors. *Immunity* **31**, 941-952 (2009).
83. J. Y. Lee, C. N. Skon, Y. J. Lee, S. Oh, J. J. Taylor, D. Malhotra, M. K. Jenkins, M. G. Rosenfeld, K. A. Hogquist, S. C. Jameson, The transcription factor KLF2 restrains CD4(+) T follicular helper cell differentiation. *Immunity* **42**, 252-264 (2015).

84. Y. S. Choi, J. A. Gullicksrud, S. Xing, Z. Zeng, Q. Shan, F. Li, P. E. Love, W. Peng, H. H. Xue, S. Crotty, LEF-1 and TCF-1 orchestrate T(FH) differentiation by regulating differentiation circuits upstream of the transcriptional repressor Bcl6. *Nat. Immunol.* **16**, 980-990 (2015).
85. A. Ulges, M. Klein, S. Reuter, B. Gerlitzki, M. Hoffmann, N. Grebe, V. Staudt, N. Stergiou, T. Bohn, T. J. Bruhl, S. Muth, H. Yurugi, K. Rajalingam, I. Bellinghausen, A. Tuettenberg, S. Hahn, S. Reissig, I. Haben, F. Zipp, A. Waisman, H. C. Probst, A. Beilhack, T. Buchou, O. Filhol-Cochet, B. Boldyreff, M. Breloer, H. Jonuleit, H. Schild, E. Schmitt, T. Bopp, Protein kinase CK2 enables regulatory T cells to suppress excessive TH2 responses in vivo. *Nat. Immunol.* **16**, 267-275 (2015).
86. A. T. Bauquet, H. Jin, A. M. Paterson, M. Mitsdoerffer, I. C. Ho, A. H. Sharpe, V. K. Kuchroo, The costimulatory molecule ICOS regulates the expression of c-Maf and IL-21 in the development of follicular T helper cells and TH-17 cells. *Nat. Immunol.* **10**, 167-175 (2009).
87. S. Dias, A. D'Amico, E. Cretney, Y. Liao, J. Tellier, C. Bruggeman, F. F. Almeida, J. Leahy, G. T. Belz, G. K. Smyth, W. Shi, S. L. Nutt, Effector Regulatory T Cell Differentiation and Immune Homeostasis Depend on the Transcription Factor Myb. *Immunity* **46**, 78-91 (2017).
88. A. Angelin, L. Gil-de-Gomez, S. Dahiya, J. Jiao, L. Guo, M. H. Levine, Z. Wang, W. J. Quinn, 3rd, P. K. Kopinski, L. Wang, T. Akimova, Y. Liu, T. R. Bhatti, R. Han, B. L. Laskin, J. A. Baur, I. A. Blair, D. C. Wallace, W. W. Hancock, U. H. Beier, Foxp3 Reprograms T Cell Metabolism to Function in Low-Glucose, High-Lactate Environments. *Cell metabolism* **25**, 1282-1293 e1287 (2017).
89. M. Herold, J. Breuer, S. Hucke, P. Knolle, N. Schwab, H. Wiendl, L. Klotz, Liver X receptor activation promotes differentiation of regulatory T cells. *PLoS One* **12**, e0184985 (2017).
90. C. Heinemann, S. Heink, F. Petermann, A. Vasanthakumar, V. Rothhammer, E. Doorduijn, M. Mitsdoerffer, C. Sie, O. Prazeres da Costa, T. Buch, B. Hemmer, M. Oukka, A. Kallies, T. Korn, IL-27 and IL-12 oppose pro-inflammatory IL-23 in CD4+ T cells by inducing Blimp1. *Nature communications* **5**, 3770 (2014).
91. R. J. Johnston, A. C. Poholek, D. DiToro, I. Yusuf, D. Eto, B. Barnett, A. L. Dent, J. Craft, S. Crotty, Bcl6 and Blimp-1 are reciprocal and antagonistic regulators of T follicular helper cell differentiation. *Science* **325**, 1006-1010 (2009).
92. T. Fu, P. Zhang, L. Feng, G. Ji, X. H. Wang, M. H. Zheng, H. Y. Qin, D. L. Chen, W. Z. Wang, H. Han, Accelerated acute allograft rejection accompanied by enhanced T-cell proliferation and attenuated Treg function in RBP-J deficient mice. *Mol. Immunol.* **48**, 751-759 (2011).
93. D. Baumjohann, R. Kageyama, J. M. Clingan, M. M. Morar, S. Patel, D. de Kouchkovsky, O. Bannard, J. A. Bluestone, M. Matloubian, K. M. Ansel, L. T. Jeker, The microRNA cluster miR-17 approximately 92 promotes TFH cell differentiation and represses subset-inappropriate gene expression. *Nat. Immunol.* **14**, 840-848 (2013).
94. N. Komatsu, K. Okamoto, S. Sawa, T. Nakashima, M. Oh-hora, T. Kodama, S. Tanaka, J. A. Bluestone, H. Takayanagi, Pathogenic conversion of Foxp3+ T cells into TH17 cells in autoimmune arthritis. *Nat. Med.* **20**, 62-68 (2014).
95. H. Wang, R. Meng, Z. Li, B. Yang, Y. Liu, F. Huang, J. Zhang, H. Chen, C. Wu, IL-27 induces the differentiation of Tr1-like cells from human naive CD4+ T cells via the phosphorylation of STAT1 and STAT3. *Immunol. Lett.* **136**, 21-28 (2011).
96. Y. S. Choi, D. Eto, J. A. Yang, C. Lao, S. Crotty, Cutting edge: STAT1 is required for IL-6-mediated Bcl6 induction for early follicular helper cell differentiation. *J. Immunol.* **190**, 3049-3053 (2013).

97. Y. Iwasaki, K. Fujio, T. Okamura, A. Yanai, S. Sumitomo, H. Shoda, T. Tamura, H. Yoshida, P. Charnay, K. Yamamoto, Egr-2 transcription factor is required for Blimp-1-mediated IL-10 production in IL-27-stimulated CD4+ T cells. *Eur. J. Immunol.* **43**, 1063-1073 (2013).
98. M. A. Koch, K. R. Thomas, N. R. Perdue, K. S. Smigiel, S. Srivastava, D. J. Campbell, T-bet(+) Treg cells undergo abortive Th1 cell differentiation due to impaired expression of IL-12 receptor beta2. *Immunity* **37**, 501-510 (2012).
99. N. Schmitt, J. Bustamante, L. Bourdery, S. E. Bentebibel, S. Boisson-Dupuis, F. Hamlin, M. V. Tran, D. Blankenship, V. Pascual, D. A. Savino, J. Banchereau, J. L. Casanova, H. Ueno, IL-12 receptor beta1 deficiency alters in vivo T follicular helper cell response in humans. *Blood* **121**, 3375-3385 (2013).
100. M. Ghoreishi, P. Bach, J. Obst, M. Komba, J. C. Fleet, J. P. Dutz, Expansion of antigen-specific regulatory T cells with the topical vitamin d analog calcipotriol. *J. Immunol.* **182**, 6071-6078 (2009).
101. I. Papa, D. Saliba, M. Ponzoni, S. Bustamante, P. F. Canete, P. Gonzalez-Figueroa, H. A. McNamara, S. Valvo, M. Grimbaldeston, R. A. Sweet, H. Vohra, I. A. Cockburn, M. Meyer-Hermann, M. L. Dustin, C. Doglioni, C. G. Vinuesa, TFH-derived dopamine accelerates productive synapses in germinal centres. *Nature* **547**, 318-323 (2017).
102. W. J. Grossman, J. W. Verbsky, B. L. Tollefsen, C. Kemper, J. P. Atkinson, T. J. Ley, Differential expression of granzymes A and B in human cytotoxic lymphocyte subsets and T regulatory cells. *Blood* **104**, 2840-2848 (2004).
103. X. Cao, S. F. Cai, T. A. Fehniger, J. Song, L. I. Collins, D. R. Piwnica-Worms, T. J. Ley, Granzyme B and perforin are important for regulatory T cell-mediated suppression of tumor clearance. *Immunity* **27**, 635-646 (2007).
104. Z. Chen, X. Luo, Y. Lu, T. Zhu, J. Wang, A. Tsun, B. Li, Ubiquitination signals critical to regulatory T cell development and function. *Int. Immunopharmacol.* **16**, 348-352 (2013).
105. M. Mandapathil, M. J. Szczepanski, M. Szajnik, J. Ren, E. K. Jackson, J. T. Johnson, E. Gorelik, S. Lang, T. L. Whiteside, Adenosine and prostaglandin E2 cooperate in the suppression of immune responses mediated by adaptive regulatory T cells. *J. Biol. Chem.* **285**, 27571-27580 (2010).
106. S. Deaglio, K. M. Dwyer, W. Gao, D. Friedman, A. Usheva, A. Erat, J. F. Chen, K. Enjyoji, J. Linden, M. Oukka, V. K. Kuchroo, T. B. Strom, S. C. Robson, Adenosine generation catalyzed by CD39 and CD73 expressed on regulatory T cells mediates immune suppression. *J. Exp. Med.* **204**, 1257-1265 (2007).
107. W. Huang, S. Solouki, N. Koylass, S. G. Zheng, A. August, ITK signalling via the Ras/IRF4 pathway regulates the development and function of Tr1 cells. *Nature communications* **8**, 15871 (2017).
108. W. Huang, A. R. Jeong, A. K. Kannan, L. Huang, A. August, IL-2-inducible T cell kinase tunes T regulatory cell development and is required for suppressive function. *J. Immunol.* **193**, 2267-2272 (2014).
109. S. S. Iyer, D. R. Latner, M. J. Zilliox, M. McCausland, R. S. Akondy, P. Penaloza-Macmaster, J. S. Hale, L. Ye, A. U. Mohammed, T. Yamaguchi, S. Sakaguchi, R. R. Amara, R. Ahmed, Identification of novel markers for mouse CD4(+) T follicular helper cells. *Eur. J. Immunol.* **43**, 3219-3232 (2013).
110. J. Azzi, N. Skartsis, M. Mounayar, C. N. Magee, I. Batal, C. Ting, R. Moore, L. V. Riella, S. Ohori, R. Abdoli, B. Smith, P. Fiorina, D. Heathcote, T. Bakhos, P. G. Ashton-Rickardt, R. Abdi, Serine

protease inhibitor 6 plays a critical role in protecting murine granzyme B-producing regulatory T cells. *J. Immunol.* **191**, 2319-2327 (2013).

Supplementary Table 2. Nanoparticles and Mouse strains

**Nanoparticles (NP)**		
NP name	*MHCII specificity*	*peptide*
Cys-capped NP	–	–
BDC2.5mi/IA^{g7}-NP	IA^{g7}	BDC2.5mi
pMOG$_{38\text{-}49}$/IA^{b}-NP	IA^{b}	MOG$_{38\text{-}49}$
**Mice**		
Strain name	*Cell specificity of Cre driver*	*Target genes*
NOD	–	–
NOD.*Scid*	–	–
NOD.*Cd4-Cre*	Pan T cell	–
NOD.*Cd4-Cre.Prdm1*$^{loxP/loxP}$	Pan T cell	*Prdm1*
NOD.*Cd4-Cre.Irf4*$^{loxP/loxP}$	Pan T cell	*Irf4*
B6	–	–
B6.*Il10*$^{loxP/mut}$	–	–
B6.*Tbx21-Cre.Il10*$^{loxP/\mathrm{mut}}$	Olfatory bulb, Th1 and Tr1 cells	*Il10*
B6.*Tbx21-Cre.Prdm1*$^{loxP/\mathrm{loxP}}$	Olfatory bulb, Th1 and Tr1 cells	*Prdm1*
B6.*Foxp3-Cre.Il10*$^{loxP/mut}$	Foxp3+ Tregs	*Il10*

Supplementary Fig. 1

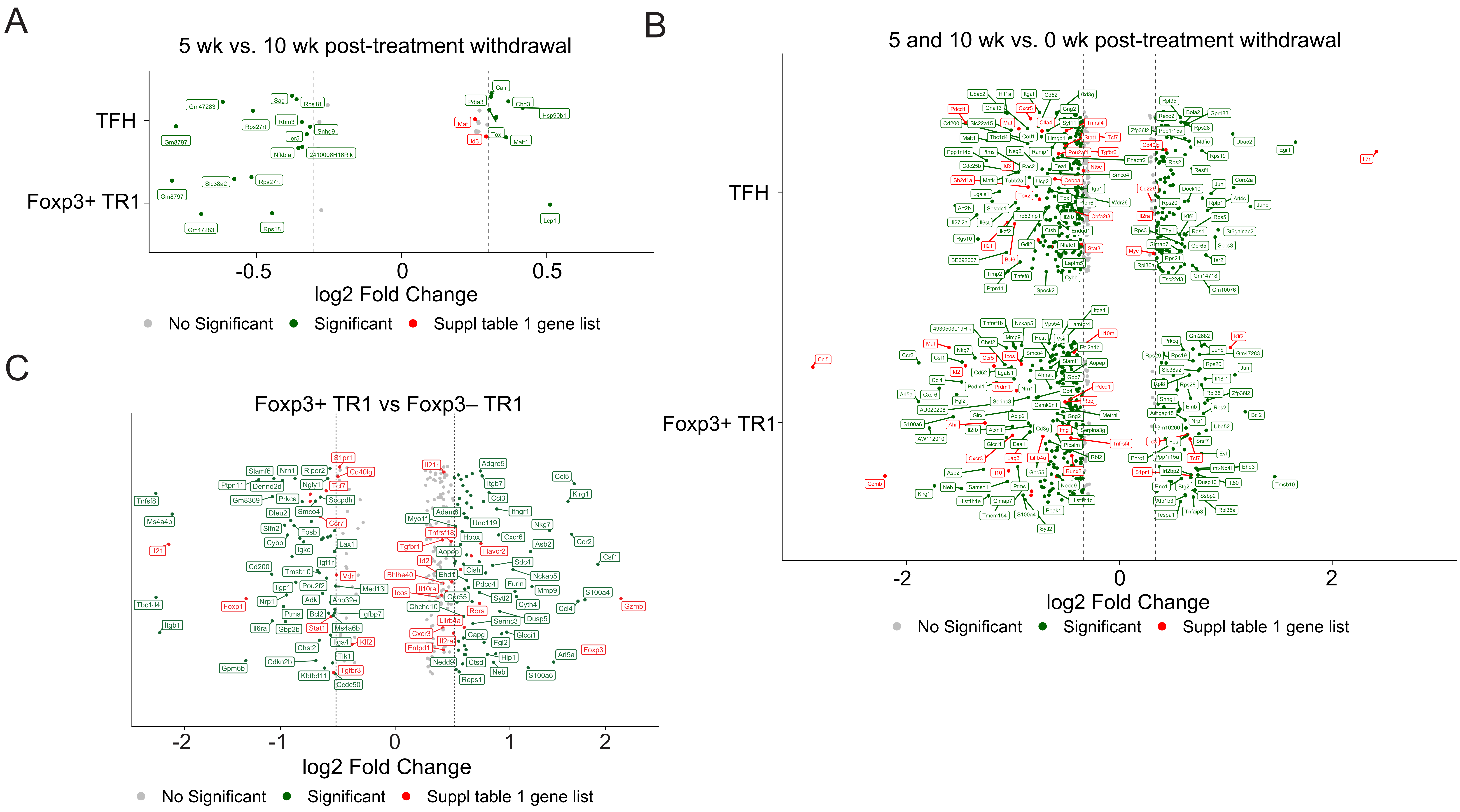

Supplementary Figure 1. **Dynamic evolution of differential gene expression in tetramer+ TFH and Foxp3+ TR1 cells post-treatment withdrawal A,** Fold change plots of differential gene expression (Wilcoxon test) between week 5 Tet+ TFH (top) and Foxp3+ TR1 (bottom) cells, respectively, and their week 10 counterparts **(Datasheet 2**). **B**, Changes in gene expression between week 5+10 Tet+ TFH (top) and Foxp3+ TR1 cells (bottom) and their week 0 counterparts **(Datasheet 3**). **C**, Fold change plot of differential gene expression using Wilcoxon test between pMHCII-NP-induced Foxp3+ TR1 and Foxp3– TR1 sub-pools. Only genes with adjusted $P < 0.05$ and $|log2FC| > 0.25$ are shown. Genes with $|log2FC| > 0.3$ (A,B) or > 0.5 (C) are shown in green, while genes with $|log2FC| <= 0.3$ (A,B) or <= 0.5 (C) are displayed in grey. Vertical lines represent –0.3/–0.5 and 0.3/0.5, respectively, of the log2FC scale. All genes listed in **Supplementary Table 1** are colored and annotated in red. Differentially expressed genes and genes listed on **Supplementary Table 1** are labeled when overlapping with less than 20 genes.

Supplementary Fig. 2

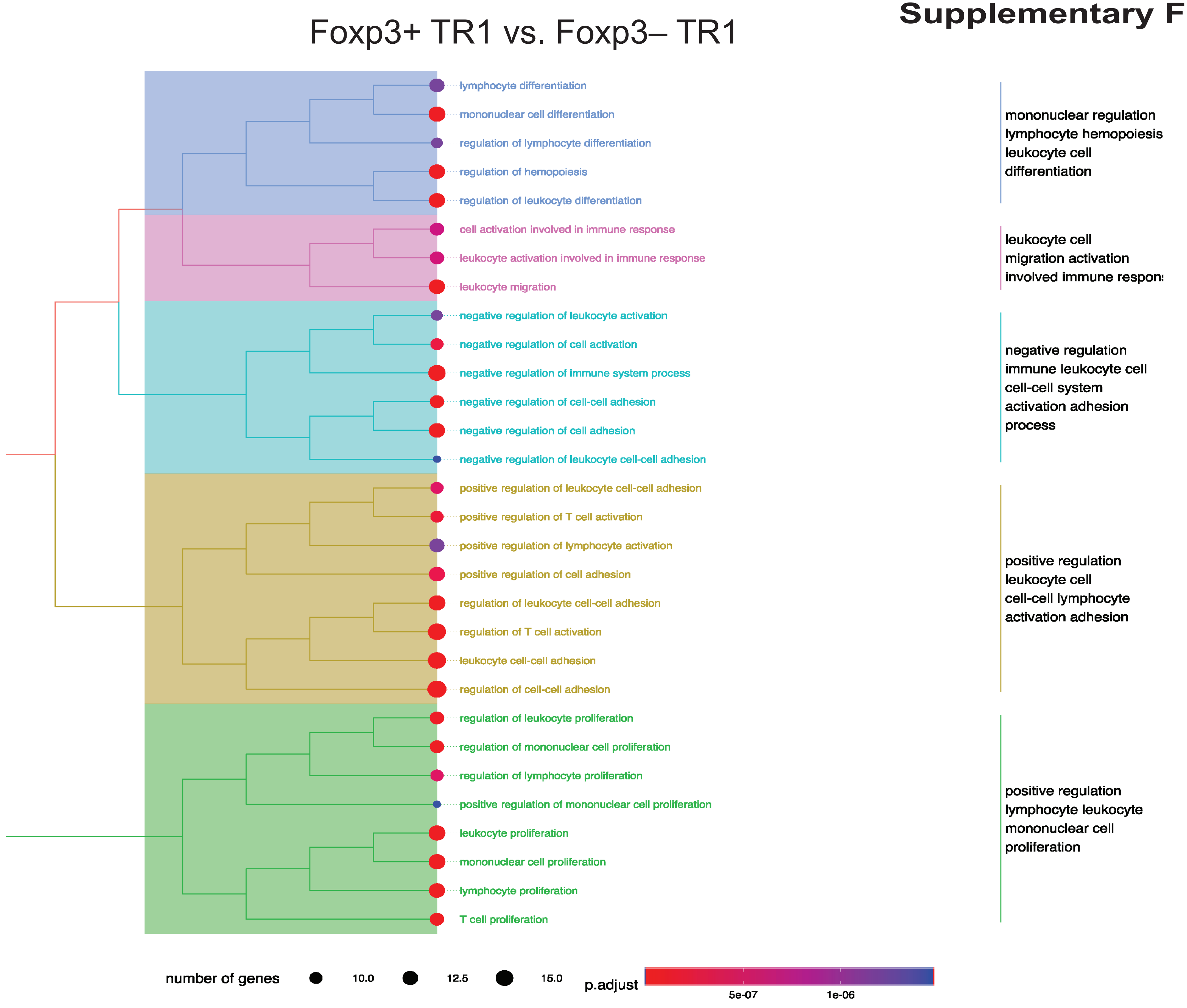

Supplementary Figure 2. **Overrepresentation comparison between BDC2.5mi/I-A^{g7}-NP-induced Tet+ Foxp3+ TR1 cells and Tet+ Foxp3– TR1 cells**. The differentially upregulated genes as defined by using the Wilcoxon test (adjusted P < 0.05 and log2FC > 0.25) were used for overrepresentation analysis, done using the Biological Process GO subontology and a q-value cutoff of 0.05. Pathways with the top 30 Gene Ratio values are shown and clustered based on common function into pathway families. Dot size represent the number of genes upregulated for each pathway and dot color the adjusted P value (p.adjust) (**Datasheet 6**).

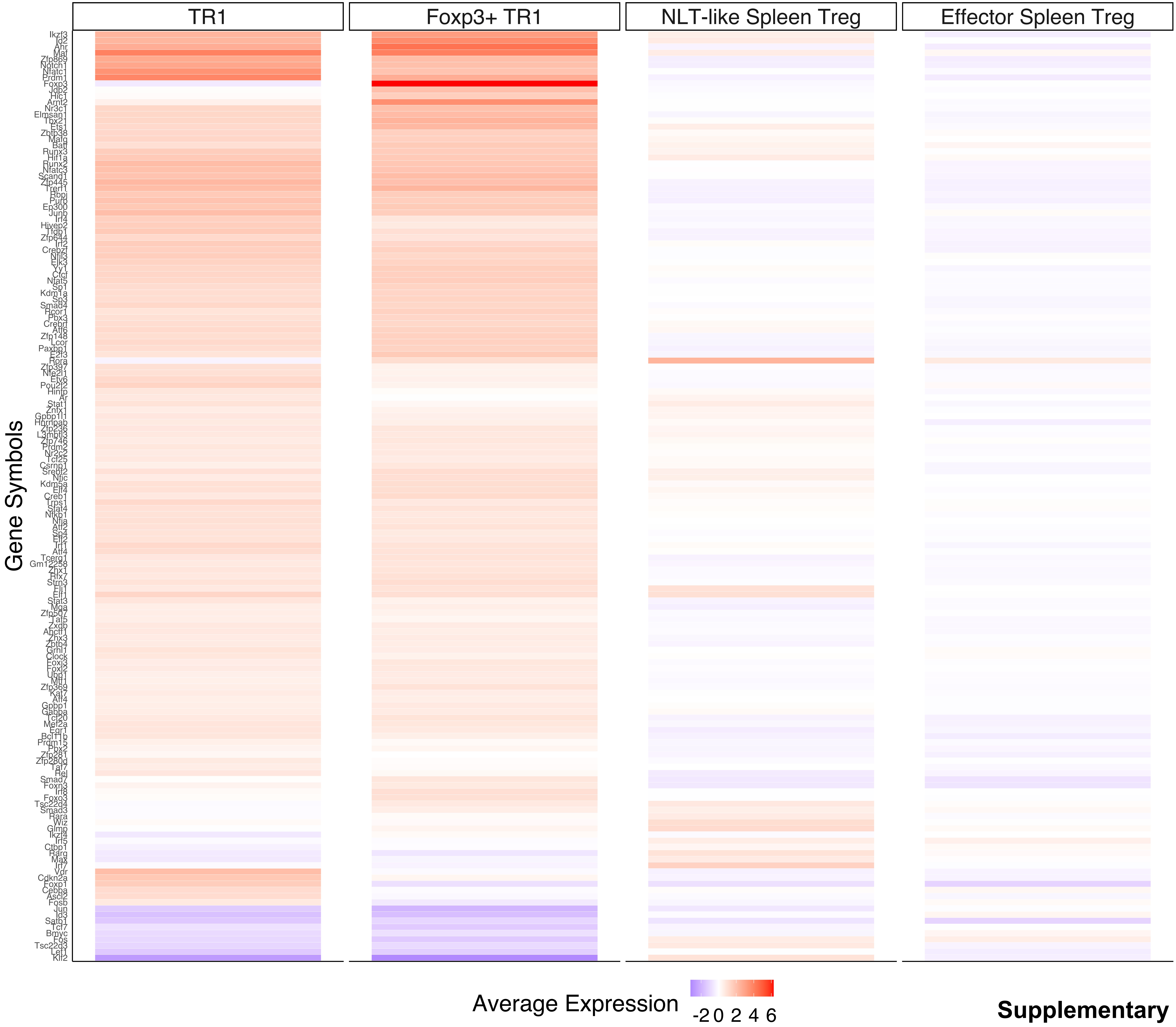

Supplementary Fig. 3

Supplementary Figure 3. Heatmap comparing scaled average expression of transcription factor coding genes (GO:0003700) that are differentially expressed in pMHCII-NP-induced pMHCII-NP-induced Foxp3+ TR1 cells vs. splenic Tregs (|log2FC| > 0.25 & adjusted P-value < 0.05) in Foxp3– TR1 cells, Foxp3+ TR1, NLT-like splenic Tregs and Effector splenic Tregs.

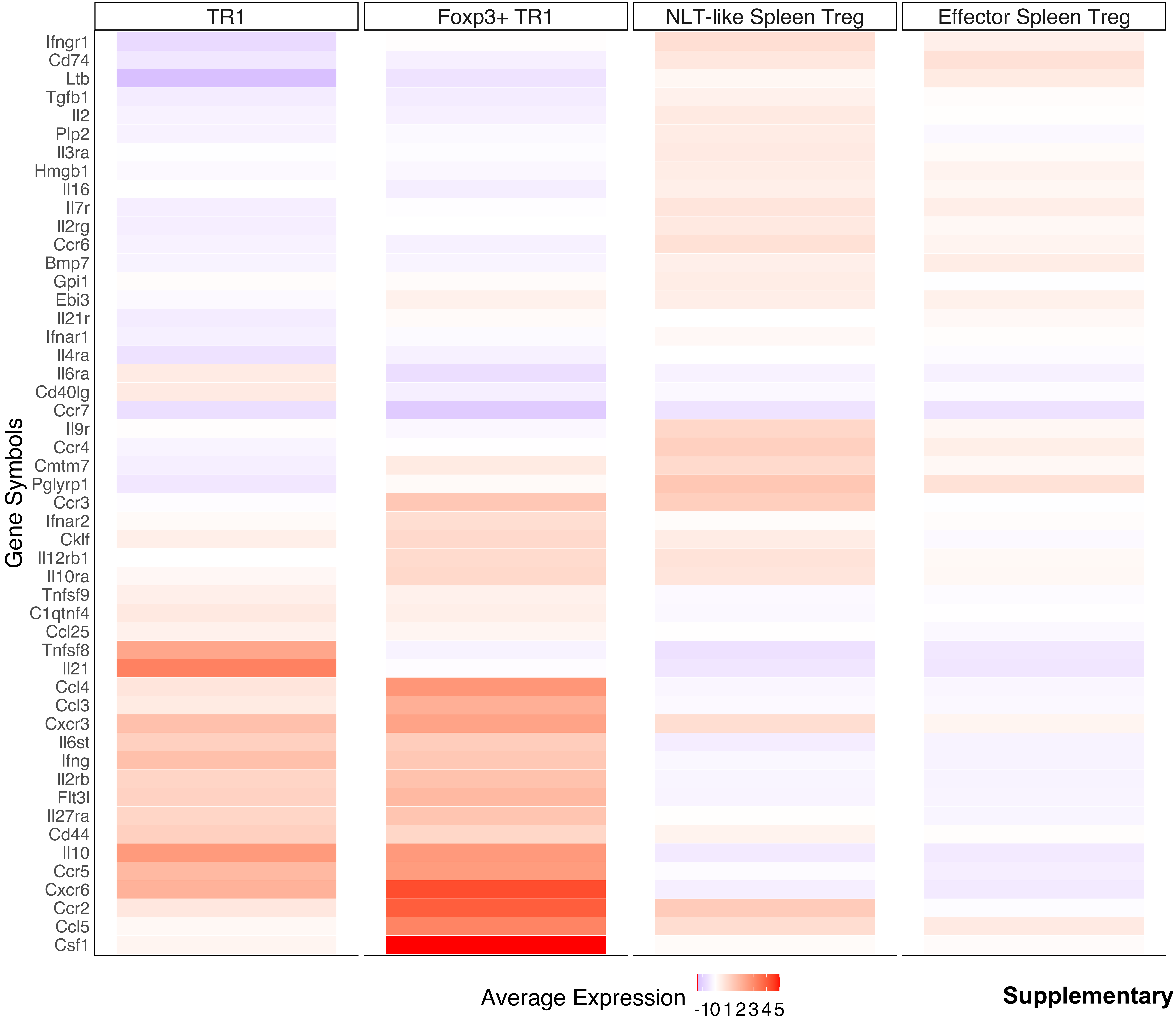

Supplementary Fig. 4

Supplementary Figure 4. Heatmap comparing scaled average expression of cytokine/chemokine and cytokine/chemokine receptor-coding genes (GO:0005125, GO:0004896, GO:0019956) that are differentially expressed in pMHCII-NP-induced pMHCII-NP-induced Foxp3+ TR1 cells vs. splenic Tregs (|log2FC| > 0.25 & adjusted P-value < 0.05) in Foxp3– TR1 cells, Foxp3+ TR1, NLT-like splenic Tregs and Effector splenic Tregs.

Suppl. Fig. 5

A

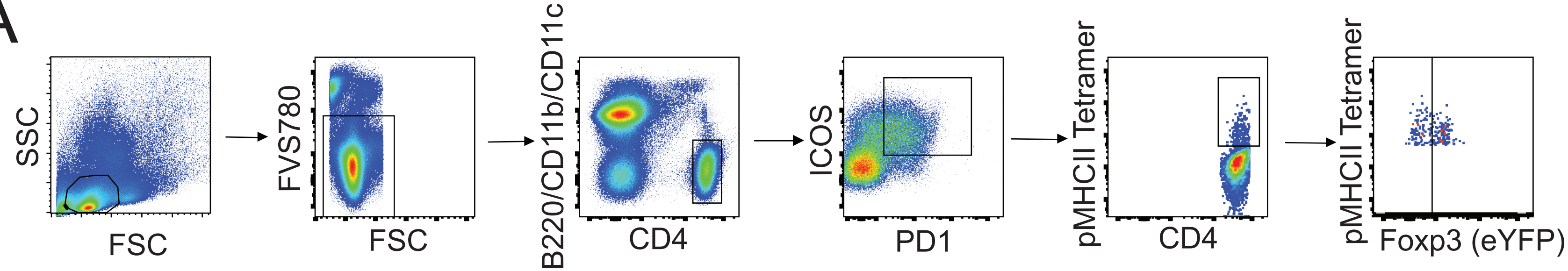
SSC
FSC
FVS780
FSC
B220/CD11b/CD11c
CD4
ICOS
PD1
pMHCII Tetramer
CD4
pMHCII Tetramer
Foxp3 (eYFP)

Supplementary Figure 5. Sorting strategy for PD1+ ICOS+ Tet+ cells from pMOG$_{38-49}$/IA^{b}–NP-treated EAE mice.

Suppl. Fig. 6

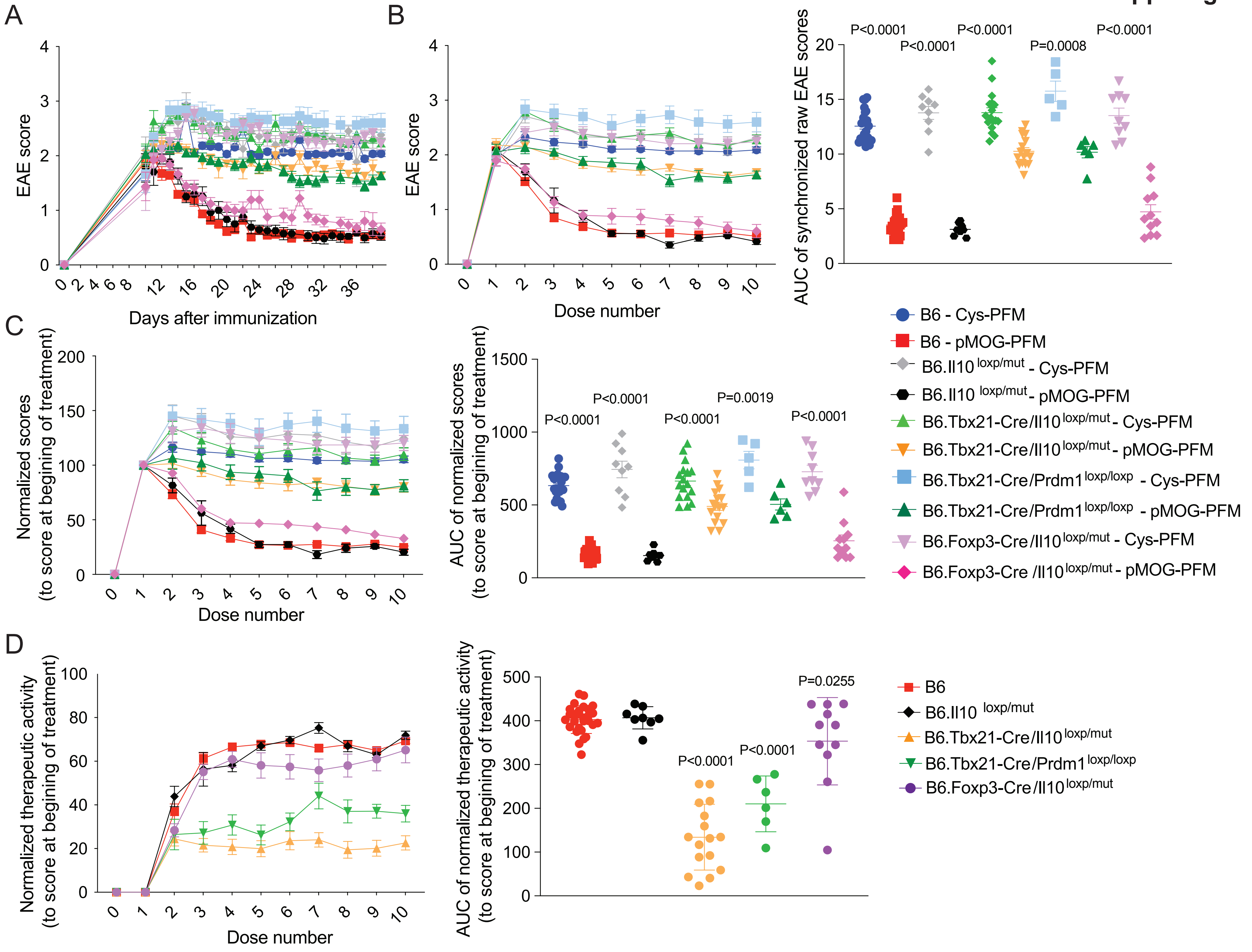
A
B
C
D
EAE score
Days after immunization
Dose number
AUC of synchronized raw EAE scores
P<0.0001
P=0.0008
Normalized scores
(to score at begining of treatment)
AUC of normalized scores
(to score at begining of treatment)
P=0.0019
Normalized therapeutic activity
(to score at begining of treatment)
AUC of normalized therapeutic activity
(to score at begining of treatment)
P=0.0255
B6 - Cys-PFM
B6 - pMOG-PFM
B6.Il10 loxp/mut - Cys-PFM
B6.Il10 loxp/mut - pMOG-PFM
B6.Tbx21-Cre/Il10 loxp/mut - Cys-PFM
B6.Tbx21-Cre/Il10 loxp/mut - pMOG-PFM
B6.Tbx21-Cre/Prdm1 loxp/loxp - Cys-PFM
B6.Tbx21-Cre/Prdm1 loxp/loxp - pMOG-PFM
B6.Foxp3-Cre/Il10 loxp/mut - Cys-PFM
B6.Foxp3-Cre /Il10 loxp/mut - pMOG-PFM
B6
B6.Il10 loxp/mut
B6.Tbx21-Cre/Il10 loxp/mut
B6.Tbx21-Cre/Prdm1 loxp/loxp
B6.Foxp3-Cre/Il10 loxp/mut

Supplementary Figure 6. **Raw EAE scores, scores normalized to the scores at the beginning of treatment, normalized therapeutic activity and the corresponding AUCs in treated mice unable to express *Il10* or *Prdm1* in Foxp3–TR1 cells or Foxp3+ cells. A,** Raw EAE scores for the strains displayed in Figure 8F. **B**, Raw EAE scores (left) and area under the curve values ((AUCs; right) for the strains on panel A, synchronized to dose number. **C**, EAE scores (left) and AUCs (right) for the strains from panel A but normalized to the scores at the beginning of treatment. **D** Left: plots of normalized therapeutic activity generated by subtracting the normalized scores (to the scores at the beginning of treatment) of Cys-NP-treated mice at each individual dose from the individual mouse EAE scores corresponding to pMHCII-NP-treated mice. Right: AUC values for the individual mouse plots used to generate the left panel. Data correspond to average $\pm$ SE of the mean. P values for AUCs between Cys-NP- and pMHCII-NP-treated mice were compared via Mann-Whitney U and corrected for multiple comparisons. P values in D were obtained by comparing values of each strain to those obtained in B6 mice via one-way ANOVA.
